# Effects of growth stage and fulvic acid on the diversity and dynamics of endophytic bacterial community in *Stevia rebaudiana* Bertoni leaves

**DOI:** 10.3389/fmicb.2015.00867

**Published:** 2015-08-25

**Authors:** Xuejian Yu, Jinshui Yang, Entao Wang, Baozhen Li, Hongli Yuan

**Affiliations:** ^1^State Key Laboratory of Agrobiotechnology, MOA Key Laboratory of Soil Microbiology, College of Biological Sciences, China Agricultural UniversityBeijing, China; ^2^Departamento de Microbiología, Escuela Nacional de Ciencias Biológicas, Instituto Politécnico NacionalMexico City, Mexico

**Keywords:** endophytes, *Stevia rebaudiana* Bertoni, bacterial diversity, pyrosequencing, growth stage, fulvic acid

## Abstract

The aim of this study was to learn the interactions among the endophytic bacteria, the plant growth, the foliar spray of fulvic acid, and the accumulation of steviol glycosides in the leaves of *Stevia rebaudiana*. Metagenomic DNA was extracted from the *Stevia* leaves at different growth stages with or without the fulvic acid treatment; and the diversity of endophytic bacteria in *Stevia* leaves was estimated by pyrosequencing of 16S rRNA genes. As results, Proteobacteria, Actinobacteria, Bacteroidetes, and Firmicutes were found to be the dominant phyla despite the growth stages and fulvic acid application. *Stevia* growth stages strongly regulated composition of endophytic community. The genera *Agrobacterium* (12.3%) and *Erwinia* (7.2%) dominated in seedling stage were apparently declined in the vegetable and initial flowering stages, while *Sphingomonas* and *Methylobacterium* increased in mature leaves at harvest time, which showed that the mature leaves of *Stevia* preferred to accumulate some certain endophytic bacteria. *Sphingomonas* and *Methylobacterium* constituted an important part of the core endophytic community and were positively correlated with the stevioside content and *UGT74G1* gene expression, respectively; while *Erwinia, Agrobacterium*, and *Bacillus* were negatively correlated with the stevioside accumulation. Fulvic acid treatment accelerated the variation of endophytes along the growth stages and increased the steviol glycosides content. This is the first study to reveal the community composition of endophytic bacteria in the *Stevia* leaves, to evidence the strong effects of growth stage and fulvic acid application on the endophytes of *Stevia*, and to demonstrate the correlation between the endophytic bacteria and the steviol glycosides accumulation.

## Introduction

Being a part of the plant associated microorganisms, endophytic bacteria live intercellular spaces or inside the plant cells (Hallmann et al., 1997; Lucero et al., 2011) at least part of their lifetime without causing visibly harmful effects on the host (Sturz et al., 2000). Although the endophytes are ubiquitous in plants, only a small fraction of the plants have been involved in the study of endophytic biology (Strobel et al., 2004; Ryan et al., 2008). As the majority of terrestrial carbon fixation and a strong biotic link between the biosphere and atmosphere, the plant leaves provide a special habitat for microorganisms, with intense solar radiation, and dramatic change of temperature (Hunter et al., 2010). Therefore, the leaves present a valuable context for investigating the relationships among the endophytic microbes, their hosts and the biotic/abiotic environmental factors (Meyer and Leveau, 2012; Zimmerman and Vitousek, 2012).

It has been reported that the endophytic communities in leaves varied with the host plant species (Yang et al., 2001) or genotype (van Overbeek and van Elsas, 2008; Hunter et al., 2010), the plant growth stage (van Overbeek and van Elsas, 2008), and the plant morphology (Elvira-Recuenco and van Vuurde, 2000; Yang et al., 2001), referring to the differences in leaf structure (shape, thickness, and stomata) and chemical properties (water and nutrients contents, secondary metabolites, Rodriguez et al., 2009; Hunter et al., 2010; Arturo et al., 2012). Furthermore, abiotic factors such as temperature, solar radiant intensity, rainfall, soil quality, and especially the fertilization have been proven to play a momentous role in regulating the modes of leaf endophytic bacterial colonization (Pedraza et al., 2009; Hunter et al., 2010). Among these factors, the plant growth stage was found overwhelming the effect of plant genotype on the total bacterial communities associated with potato (van Overbeek and van Elsas, 2008). In addition, it has been proved that the plant growth regulators could simulate lots of physiological processes in plants and improve the plant performance (Ren et al., 2011; Luczkiewicz et al., 2014). However, little information is available about the effects of growth regulators on the endophytes associated with plant leaves.

*Stevia rebaudiana* Bertoni is an important economic plant for producing steviol glycosides (SGs), a kind of natural, non-caloric, high-intensity sweeteners approved as natural sweeteners beneficial to health (Yadav et al., 2011). In China, this plant has been extensively cultivated in last decades as the third most popular natural sugar source, just after cane and beet (Yang et al., 2013). In *Stevia* leaves, eight kinds of SGs have been detected, with stevioside (ST), rebaudioside A (RA), and rebaudioside C (RC) as the major ones (Yadav et al., 2011), in which RC has an undesirable bitter aftertaste, which restricts its application in the food industry for human direct consumption; while RA has a higher sweetness rating and better taste than RC. The SGs are accumulated in the *Stevia* leaves and their concentration varies widely depending on the genotype, fertilization level and growth stages (Yadav et al., 2011; Yan et al., 2012) via their effects on the 17-step biosynthetic pathway of SGs (Kumar et al., 2012; Madhav et al., 2013; Chen et al., 2014). For the biosynthesis of SGs in *Stevia*, the last five steps are specific and catalyzed by at least three UDP-glycosyltransferases genes: *UGT85C2* gene responsible for the addition of C-13-glucose to steviol; *UGT74G1* responsible for addition of glycosyl to steviolbioside; and *UGT76G1* coding the key enzyme for transferring a glucose residue to the ST molecule for synthesizing RA (Supplementary Figure S1) (Kumar et al., 2012; Madhav et al., 2013; Chen et al., 2014). Thus, enhancing the expression level of the *UGT* genes through certain treatments might be a possible way to increase the RA content in the *Stevia* leaves. Previously, we found that the spread of fulvic acid (FA), an eco-friendly plant regulator (Nardi et al., 2002), could increase *Stevia* leaf biomass. However, the biological mechanism of this effect was unclear. Considering the economic importance of *S.rebaudiana* and the special characters of its leaves (high RA content), these plants could be considered as a valuable model for investigating the diversity and dynamics of endophytic bacteria in leaves, and for evaluating the interactions among the endophytic community, the metabolites accumulation and the application of growth regulator. Therefore, we performed this study to compare the endophytic bacterial communities in *Stevia* leaves with or without plant regulator (FA) treatment at different growth stages, using the 454 pyrosequencing of 16S rRNA gene. In addition, we also investigated the relationships among the endophytic communities, the SGs content and *UGT* genes expression. The aim was to provide a potential insight into the plant-microorganism interactions in *Stevia*. It was the first study on the endophytic bacterial community in *Stevia* leaves and on the correlation between the endophytes and the accumulation of SGs.

## Materials and methods

### Plant growth conditions and treatments

Rooted plantlets of a high-RA-yielding variety of *Stevia rebaudiana* Bertoni, Xinguang No. 3, purchased from Lvyuan *Stevia* Co. Ltd. (Mingguang, Anhui, China) were transplanted in plots (20 m^2^ for each) in the fields at Shangzhuang experimental station of China Agricultural University (116.18°E, 40.14°N) in May 8 of 2012. The experiment consisted of a randomized block design with four biological replications for the control and the FA treatments, respectively; therefore, a total of eight plots were included. The described field study did not require specific permits and did not involve endangered or protected species.

The plants were put in the plots with ~30 cm of space between plants in the rows and with distance of 45 cm between two rows. Urea (15 g m^−2^) was supplied as the basic fertilizer in soil for all the experiments. Treatment F was applied with a foliar spray of FA solution (500 mg l^−1^) at the dose of 75 ml m^−2^ once every 2 weeks after transplanting; while water was applied for the control group. The FA solution was obtained from the biodegradation of leonardite with the bacterial community MCSL-2 (Gao et al., 2012) by centrifugation at 8000 × *g* for 15 min and filtered through Whatman No. 1 to remove cells and residual leonardite after 21 days of incubation. The supernatant was dried at 60°C and dissolved in water at the concentration of 500 mg l^−1^ (Gao et al., 2012).

### Leaf sampling and metagenomic DNA extraction

In this analysis, *Stevia* plants from control and FA treatments were collected at three growth stages: the seedling stage (coded as C-0), the vegetable growing stage (after 2 months of growth, coded as C-2 for control and F-2 for FA treatment), and the initial flowering stage (after 4 months of growth, coded as C-4 for control and F-4 for FA treatment). From each plot, 10 randomly selected plants were uprooted. The leaves of sampled plants were cut off immediately using a razor blade and all the leaves from the same plot were stored in a paper bag as a single sample. During all the sampling procedure, sterile gloves were used by the workers to avoid bacterial contamination. The samples were transported to the laboratory on ice, and then stored at −80°C before further processing.

To isolate the metagenomic DNA, all the sampled leaves were surface cleaned to reduce the presence of surface microorganisms by the following procedure: simply rinsed in sterilized deionized H_2_O; immersed in 70% (v/v) ethanol (2 min); immersed in sterilized deionized H_2_O (2 min); immersed in 5% (w/v) NaClO (3 min), and finally received three sequential 1 min rinses in sterilized H_2_O (Bodenhausen et al., 2013). The leaves were dried at 37°C for 4 h and then the samples from the same treatments at the same stage were compiled and ground in liquid nitrogen. A modified endophytic DNA enrichment method (Wang et al., 2008b) was employed to avoid the influence of leaf chloroplast DNA. The metagenomic DNA was extracted from 0.5 g of each enriched endophytic bacterial sample using an E. Z. N. A.TM Soil DNA Kit according to the manufacturer's instructions (Omega Bio-tek, Inc., USA).

### PCR amplification and pyrosequencing analysis

Primer set 515F/926R was used to amplify the 16S rRNA gene fragment (V4 and V6 region) from the samples on the 454 GS-FLX pyrosequencing platform and duplicate was employed for each sample. The universal primer set 515F/926R specific to V4 and V5 regions and PCR protocol describe previously and the reverse primer included a 7 bp barcode (Quince et al., 2011). The PCR conditions used were 95°C for 5 min, 30 cycles of 95°C for 40 s, 55°C for 40 s, and 72°C for 1 min, followed by 72°C for 7 min (Andersson et al., 2008; Lopez-Velasco et al., 2011). The sequencing data were processed using the Quantitative Insights Into Microbial Ecology (QIIME) software (Caporaso et al., 2011), and suspected chimeras were detected using the QIIME chimera with a denoising step. To compare the samples, sequences below a quality score of 25 and <200 bp were removed, and the adapters, barcodes and primers were trimmed using default parameters. Sequences representing chloroplast or mitochondrial DNA were eliminated from further analysis (Bodenhausen et al., 2013). The obtained sequences were assigned into operational taxonomic units (OTUs) using 97% identity clustering, and the most abundant sequence from each OTU was selected as the representative sequence for that OTU. Taxonomy was assigned from the RDP database with 80% confidence. Sequences were assigned to phylotype clusters at two cut-off levels of species, 3 and 5%. Based on the sequences and/or OTUs obtained, rarefaction curves, ACE, Chao1 richness and the Shannon index were calculated. UPGMA clustering analysis, PCoA, CCA were performed to evaluate the similarities and to correlate the microbial distribution with the environmental factors for the five samples. The pyrosequencing reads have been deposited at the GenBank under accession number SRR1552085.

### Determination of biomass yield and content of SGs in *Stevia* leaves

After 4 months growth, the control and FA treated plants were harvested separately for each plot, and the leaves obtained from each plot were dried in an oven at 50°C to a constant weight. The dried leaves from each plot were then weighted and ground separately using a high-speed grinder. An aliquot of 0.25 g of the ground leaves from each sample was extracted in a 150-mL Erlenmeyer flasks with 25 mL of 70% (v/v) ethanol in a 70°C water bath by shaking (150 rpm) for 30 min (Moraes et al., 2013; Serfaty et al., 2013). After cooling, aliquot of 1 mL was filtered with 0.22 μm filter (Beihua Sunrise Barrier Separation Technology, Beijing, China) and 20 μL of the filtrated extract were analyzed by high-performance liquid chromatography (HPLC; Shimadzu Essentia LC-15C, Shimadzu Corporation, Kyoto, Japan) using a Phenomenex Luna-NH_2_ column (5 μm, 250 × 4.6 mm; Phenomenex Inc., Torrance, CA, USA). A mixture of acetonitrile and water (75:25, v/v) was used as the eluent at a flow rate of 1 mL min^−1^, and the compounds were detected with ultraviolet (UV) light at 210 nm. For quantitative analysis, pure ST and RA obtained from Liaoning Qianqian biotechnology Co., Ltd were separately used to prepare the standard solutions in ethanol at the concentrations of 0.3, 0.6, 0.9, and 1.2 g l^−1^. The parameters of external calibration curves were obtained by fitting experimental data through linear regression from replicate injections of standard solutions (Kolb et al., 2001).

### *UGT* genes expression

In this assay, 10 plants from each plot were randomly selected and 5 g of the leaves were mixed as one sample for RNA isolation (Brandle et al., 2002; Madhav et al., 2013) with TRI gene reagent (Genstar), according to the manufacturer's instructions. After quality control, 1 μg of the high purity RNA samples (A_260∕280_ > 1.8) was used for cDNA synthesis with PrimeScript cDNA Synthesis Kit (Takara, Japan) following the manufacturer's instructions.

For real-time quantitative polymerase chain reaction (RT-q PCR), the β*-actin* and 18S rRNA genes, which are expressed at a constant level across all samples, were used to normalize the data individually for more reliability (Mohamed et al., 2011). Primers for both *UGT*, β*-actin* and 18S rRNA genes were designed according to Mohamed et al. (2011) (sequences in 5′–3′, the annealing temperature of the primers was listed in the parenthesis):

*UGT76G1* (60°C):f-GCAGCTTACTAGACCACGATC*r*-CTCATCCACTTCACTAGTACTAC
*UGT74G1* (60°C):f-TGCATGAACTGGTTAGACGATAAG*r*-GCATCCTACTGATTCGTGTGCTA
*UGT85C2* (60°C):f-TCGATGAGTTGGAGCCTAGTATT*r*-CTAAACTGTATCCATGGAGACTC
β*-actin* (60°C):f-AGCAACTGGGATGACATGGAAr-GGAGCGACACGAAGTTCATTG
18S rRNA (60°C):f-CCGGCGACGCATCATTr-AGGCCACTATCCTACCATCGAA

The PCR reaction was performed in 20 μl containing 8.2 μl H_2_O, 10 μl SYBR green mix, 0.4 μl of each primer (10 μmol l^−1^) and 1 μl of cDNA obtained as mentioned above. The reactions were applied as quadruplicates in an ABI PRISM 7500 sequence detection system (ABI, Applied Biosystems) with the following thermal cycles: 95°C for 10 min, followed by 40 cycles of 95°C for 30 s and 60°C for 30 s.

### Statistical analysis

To estimate the effects of FA treatment, the data of leaf yields and contents of SGs were statistically analyzed using the software package of Microsoft Excel and SPSS 17.0 for Windows (IBM Corp., Armonk, NY, USA). To examine the statistical significance of differences between treatment and control groups, a One-Way analysis of variance (ANOVA) was conducted through the Tukey HSD-test.

## Results

### Overall diversity of the endophytic bacteria in *Stevia* leaves

After quality control, a total of 49,289 sequences were obtained from all the five samples in the pyrosequencing analysis (Table 1). Excluding the potential chimeras, 2164 bacterial OTUs were obtained from the leaf endophytic samples, at the 0.03 distance cutoff. The average number of OTUs observed in the samples was 690.8 (*SD* = 101.5). The coverage parameters OTUs/Chao1 and OTUs/ACE were about 60 and 50%, respectively. The species richness (OTU numbers and Simpson index) of leaf endophytes was apparently increased along the growth of plant: 38.6 and 29.9% greater than that in C-0 after 2 (C-2) and 4 (C-4) months growth, respectively, as shown in Table 1. However, the Shannon index was decreased. In comparison with the control samples (C-2 and C-4), the fulvic acid treatment (F-2 and F-4) decreased both the species richness and the diversity index: −23.8 and −18.2% for OTU number; −1.76 and −0.9 for Shannon index, respectively.

**Table 1:** **General data of pyrosequencing results of leaf endophytic bacteria and growth/quality characters of ***Stevia*** in different growth stage and treatments**.

**Characters**	**Growth stage and treatment^#^**				
	**C-0**	**C-2**	**C-4**	**F-2**	**F-4**
**PYROSEQUENCING DATA**					
Reads	3670	10,228	10,752	8210	16,429
OTUs	595	825	773	629	632
Chao1^§^	1073	1256	1467	928	1006
ACE^¶^	1492	1264	1900	1219	983
Shannon	6.68	4.73	5.24	2.97	4.34
Simpson	0.032	0.211	0.101	0.461	0.163
GROWTH AND QUALITY^*^					
Plant height (cm)	12.60 e	38.10 d	89.40 b	41.9 c	99.00 a
RA content (%)	8.08 c	10.20 b	11.95 ab	13.20 a	13.65 a
ST content (%)	0.50 c	0.80 bc	1.77 ab	0.97 bc	2.28 a
RC content (%)	0.23 c	0.96 ab	1.42 a	0.70 bc	1.31 a
SGs content (%)	8.81 c	11.96 b	15.14 a	14.87 a	17.24 a
76G1 gene expression	1.00 c	0.46 d	0.16 e	2.04 b	4.34 a
85C2 gene expression	1.00 b	0.50 c	0.04 d	2.52 a	0.25 cd
74G1 gene expression	1.00 d	9.40 ab	3.67 c	10.49 a	8.52 b

* *Data were average of four replicates and numbers in the same line followed by different letters presented significant difference at P = 0.05*.

# *C, control; F, fulvic acid treatment; 0, seedling stage; 2, middle vegetable growth stage; 4, initial flowering stage*.

§ *Calculated at a distance level of 0.03*.

¶ *Ninety-five percent confidence interval. ACE, abundance-based coverage estimators*.

**Figure 1:**
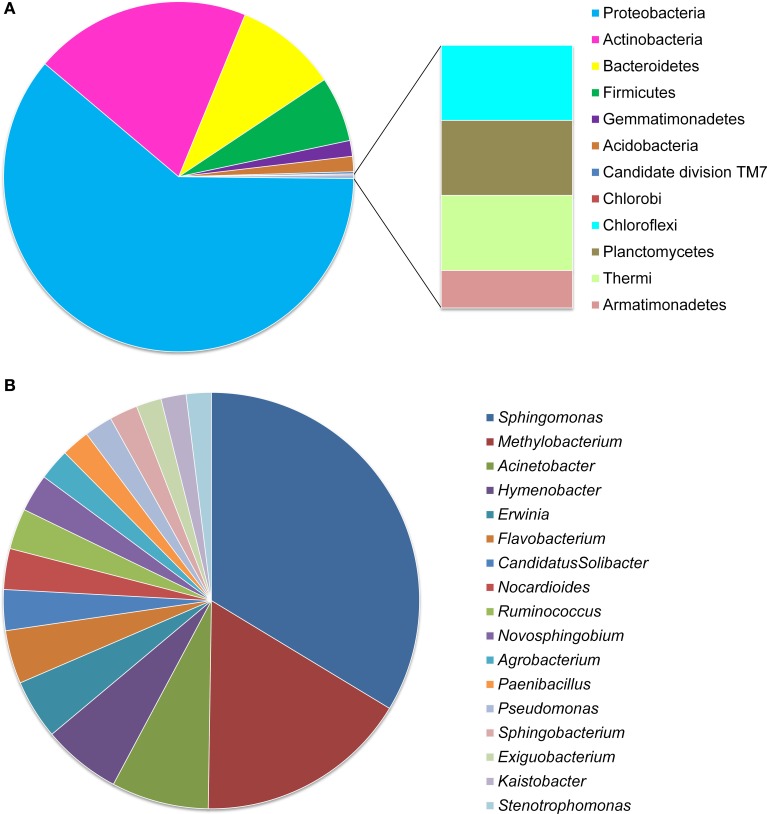
**OTU distribution of ***Stevia*** leaf endophytic bacteria at phylum (A) and genus (B) levels**. Data were obtained from all the five samples of the two treatments and three growth stages.

**Figure 2:**
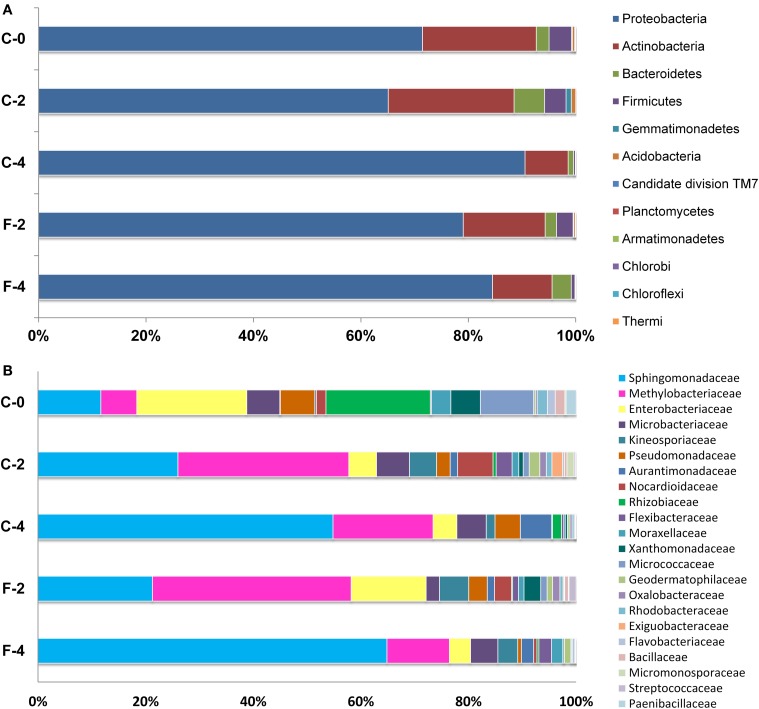
**Relative abundance of bacterial phyla (A) and families (B, taxa represented occurred at >1% abundance in at least one sample) associated with ***Stevia*** leaves as determined from pyrosequencing**. Percentages represent the portion of 16S rRNA gene 454 reads which were classified to that phylum and family.

Among these OTUs, 2048 (94.6%) were identified into 12 phyla and only six of them presented an average abundance >1% of the OTUs (covering 0.24–79.32% of reads) (Figures 1A, 2A, details available as Supplementary Table S1): 58.09% of OTUs covering 79.32% of reads were Proteobacteria; 19.09% of OTUs corresponding to 14.32% of reads were Actinobacteria; 9.01% of OTUs with 3.09% of reads were Bacteroidetes; 5.73% of OTUs with 1.94% of reads were Firmicutes; 1.39% of OTUs covering 0.24% of reads were Gemmatimonadetes; 1.34% of OTUs with 0.30% of reads were Acidobacteria, and 1–4 OTUs (<0.2%) covering 2–7 (≤0.014%) of reads belonged to each of the Candidate division TM7, Chlorobi, Chloroflexi, Planctomycetes, Thermi, and Armatimonadetes. At the family level, 71.49% of OTUs were identified into 131 taxa, with Sphingomonadaceae (15.26% for OTUs and 41.59% for reads) and Methylobacteriaceae (8.02% for OTUs and 19.19% for reads) as the most abundant ones, followed by Enterobacteriaceae (6.59% for OTUs and 6.52% for reads), Microbacteriaceae (3.30% for OTUs and 4.69% for reads), and Kineosporiaceae (0.97% for OTUs and 3.26% for reads); while each of the other families occupied <3% of the reads (Figure 2B, Supplementary Table S2).

At the genus level, only 813 (37.6%) OTUs were identified into 187 taxa (Figure 1B, Supplementary Figure S2), including *Sphingomonas* (16.97% for OTUs and 36.77% for reads) and *Methylobacterium* (8.36% for OTUs and 11.62% for reads) as the most abundant ones, followed by the genera *Acinetobacter* (3.81% for OTUs), *Hymenobacter* (3.08%), *Pseudomonas* (2.58%), *Erwinia* (2.34%), *Flavobacterium* (1.60%), *Nocardioides* (1.60%), and *Paenibacillus* (1.11%) etc.; while each of the other genera occupied <1.25% of the reads (Figure 1B, Supplementary Figure S2 and Supplementary Table S3).

### Variation of endophytic bacterial communities along growth stage

**Figure 3:**
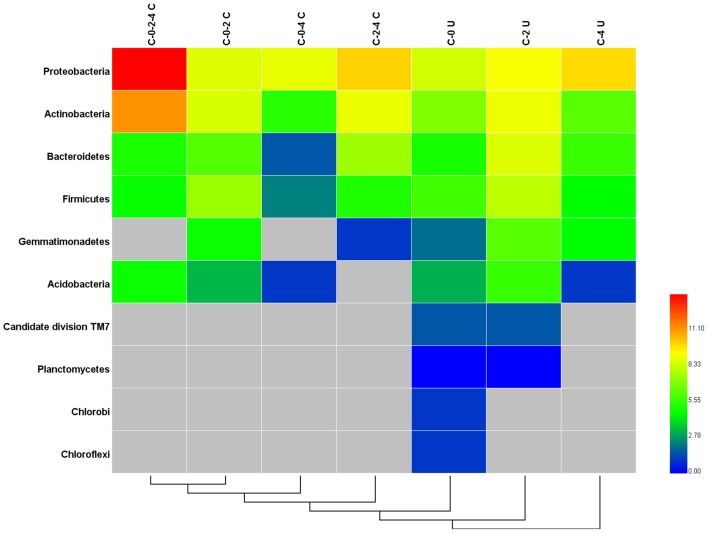
**Heatmap of the shared and unique phyla in ***Stevia*** leaf samples at different growth stages**. C-0-2-4 C: common phyla for stages C-0, C-2, and C-4; C-0-2 C: common phyla for stages; C-0-4 C: common phyla for stages C-0 and C-4; C-2-4 C: common phyla for stages C-2 and C-4; C-0 U: unique phyla for stage C-0; C-2 U: unique phyla for stage C-2; C-4 U: unique phyla for stage C-4.

The variation of community composition in leaf endophytes was estimated at the levels of OTU (Supplementary Figure S3), phylum, and genus (Figure 2A, Supplementary Figure S2). Among the 1700 OTUs, only 96 (5.65%) were found in all the three growth stages (samples C-0, C-2, and C-4) (Supplementary Figure S3), which comprised 71.61% of the total pyrosequencing reads and formed the core microbiome of the leaf endophytes for *Stevia*. Among the core microbiome, Proteobacteria (85.72%), and Actinobacteria (13.77%) are the dominant groups, followed by Bacteroidetes, Acidobacteria, and Firmicutes, which occupied less than 0.20% of the reads (Figure 3, Supplementary Table S4). Furthermore, 43 genera, dominated by *Sphingomonas, Methylobacterium, Acinetobacter, Bacillus, Nocardioides*, and *Pseudomonas*, were found in the core microbiome (Supplementary Table S3). Similar core microbiome was also found in the fulvic acid treatments (F-2 and F-4).

The change of relative abundance for each of the phyla, especially the six principle ones mentioned above was obvious in the three growth stages (Figure 3). Many of the stage specific OTUs were found as rare and high diverse sequences. The C-0, C-2, and C-4 samples harbored 337 (19.82%), 499 (29.35%), and 467 (27.47%) unique OTUs, respectively (Supplementary Figure S3). These unique fractions comprised a major proportion (76.65%) of OTUs, but merely proportion (14.70%) of the reads. The stage specific OTUs were dominated by the Proteobacteria (5.3%), Actinobacteria (2.0%), Bacteroidetes (1.3%), and Firmicutes (1.0%), in which the proportions of Bacteroidetes and Firmicutes were much higher than those in the common OTUs (Figure 3). Candidate division TM7, Planctomycetes, Chlorobi, Chloroflexi, and Gemmatimonadetes were specifically presented in the unique OTUs and the high variety of stage specific OTUs lead to the differences among the community structure of endophytic bacteria in various growth stages.

At the family level, different trends of species abundance were observed in the three growth stages (Figure 2B, Supplementary Table S2). Enterobacteriaceae (18.43%) was the most abundant family followed by Rhizobiaceae (17.46%), Sphingomonadaceae (10.52%), Micrococcaceae (8.92%), and Methylobacteriaceae (6.01%) in sample C-0. In the sample C-2, Methylobacteriaceae (27.68%) and Sphingomonadaceae (22.67%) were the dominant families; while in the sample C-4, the most abundant families were Sphingomonadaceae (53.20%), followed by Methylobacteriaceae (18.04%). The relative abundance of different families varied from stage to stage. The dominated taxa shifted dynamically along the growth stage: the relative abundance of Enterobacteriaceae and Rhizobiaceae fell from 18.43 and 17.46% in C-0 to 4.3 and 1.6% in C-4, respectively. On the other hand, the abundance of Sphingomonadaceae showed a substantial increase from 10.5% at seedling stage to 22.7% at the vegetable stage, and then to 53.2% at flowering stage. Compared to the samples of C-2 and C-4, Micrococcaceae was much more enriched in the C-0 sample and the relative abundance reached to 8.92% of the reads while the average abundance in the other two samples was <1%. Overall, the taxonomic distribution of leaf endophytes in C-0 was much more scattered than those in C-2 and C-4. In other words, the community composition of endophytes in mature leaves was more centralized and the two most abundant families comprised over 50 and 70% reads in C-2 and C-4, respectively.

The community composition at the genus level was consistent to that at the family level (Supplementary Figure S2). Many of the identified genera, like *Actinomadura* in the seedling stage (C-0), *Agromyces* in the vegetable stage (C-2), *Hyphomonas* in the initial of flowering stage (C-4), were recorded.

### Effects of fulvic acid on production and endophytic communities of *Stevia*

In this study, the final dry weight of *Stevia* leaves was significantly increased (26.2%, *P* <0.05) in the fulvic acid treatment (2801.2 kg ha^−1^) compared with that of the control (2219.5 kg ha^−1^). Similar effects were also detected in the plant height, *UGT* gene expression and contents of RA and ST in the FA treatment (Table 1, Supplementary Figure S4). Corresponding to the increases in leaf biomass and plant height, the community composition of the leaf endophytes was also dramatically modified by the FA treatment. The data of OTU numbers and indices of Chao1, ACE and Shanon (Table 1) showed a clear decrease in diversity of endophytes. The shifting in community composition caused by the FA treatment can be observed from Figure 2 (Supplementary Tables S1, S2). FA treatment reduced the abundances of Sphingomonadaceae, Microbacteriaceae, Comamonadaceae, Nocardiodaceae, but increased the abundances of Methylobacteriaceae, Psuedomonadaceae, Enterobacteriaceae, and Xanthomonadaceae in the vegetable growth stage. In the initial flowering stage, the abundances of Sphingomonadaceae and Kineosporiaceae were increased; companying with the decrease in abundances of Methylobacteriaceae, Aurantimonadaceae, and Psuedomonadaceae. These effects can also be found at the genus level, such as presence of *Aerococcus* and absence of *Aquicella* in the FA treatment (Supplementary Table S3).

### PCoA/CCA analysis of endophytic communities

**Figure 4:**
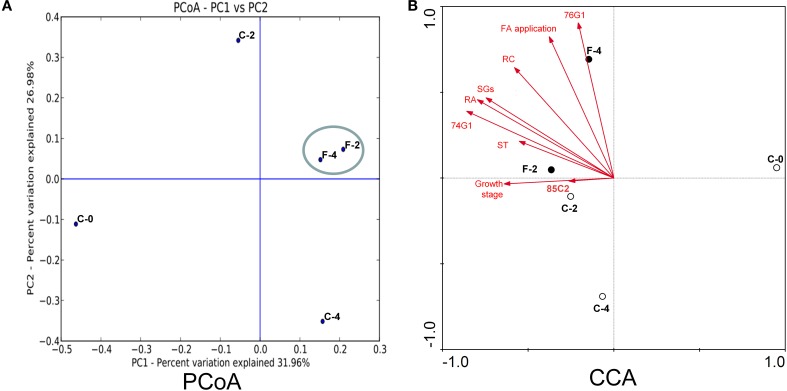
**PCoA (A) and CCA (B) analysis of the endophytic samples in ***Stevia*** leaves with different growth stages with or without FA treatment**.

The PCA analysis revealed that the growth stage was a strong interpretive factor for the variation in community composition of the leaf endophytes (Figure 4A). The first principal coordinate separated the samples based on the growth stage, the seedling stage clearly separated from the other growth stages along PC1 which showed an obvious difference among the samples. Samples at the same growth stage with or without FA treatment (C-2/F-2, C-4/F-4) were separated from each other, and the FA treated samples formed a close cluster, indicating that the FA treatment was also a factor to shape the community composition.

**Figure 5:**
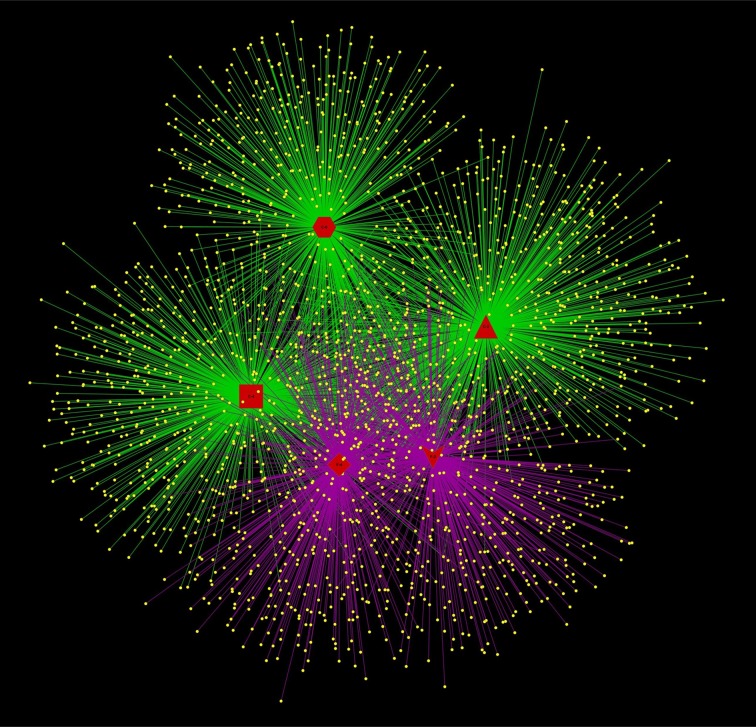
**Networks representing sample/OTU interaction**. OTU nodes are yellow, with edges indicated according to FA treatment (purple: with FA treatment; green: without FA treatment). Sample nodes are shown in red color with different shapes according to growth time and FA treatment (Hexagon, C-0; Triangle, C-2; Rect, C-4; Vee, F-2; diamond, F-4).

The relevance of the OTUs for community composition was better explained through the networks (Figure 5), which was characterized by edges linking the OTUs (shown in yellow) with the corresponding samples (shown in red). The distribution of sample nodes in the networks highlighted the separation according to growth stage and FA treatment. The samples treated by FA were clustered closer and shared more common OTUs than the controls, proving again that the FA treatment was highly involved in shaping the endophytic bacterial communities in *Stevia* leaves.

The relationship between leaf endophyte community structure and biotic/abiotic factors (steviol glycosides content, growth stage, *UGT* gene expression, and FA application) was revealed by the canonical correspondence analysis (Figure 4B). The first two CCA axes explained as high as 57.0 and 28.3% of the total variance in the bacterial community data, and significant species–environment correlations were observed, indicating that the CCA result was reliable. According to CCA analysis, the *UGT74G1* gene expression, contents of RA and SGs were best correlated with axis 1 (*r* = −0.8588, −0.7963, −0.7461, respectively), while *UGT76G1* gene expression and FA application were best correlated with axis 2 (*r* = 0.9057, 0.8238, respectively).

### Relationship between the core community and plant performances

**Figure 6:**
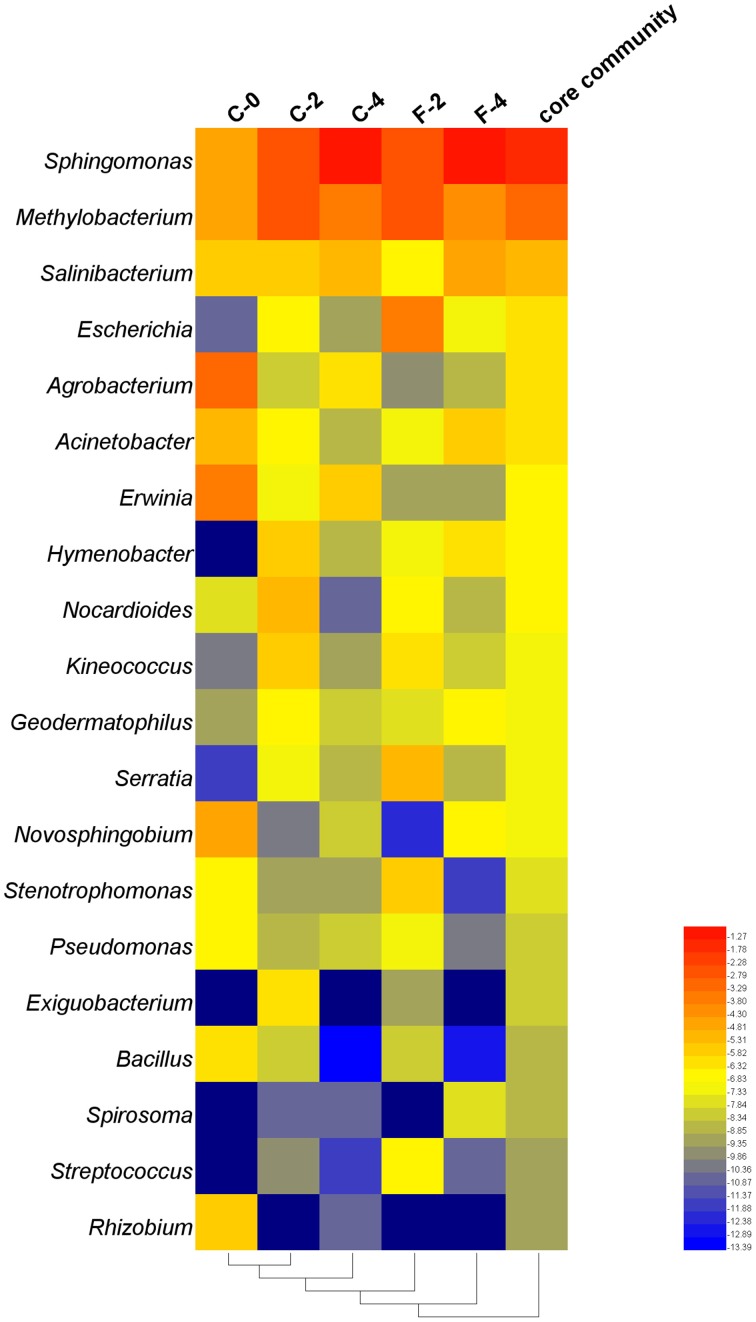
**Heatmap of taxonomic distribution of the core community of the endophytic samples in ***Stevia*** leaves with different growth time at the genus level**.

To construct a core bacterial community of *Stevia* leaf endophytes, we pooled the 10 most abundant genera in each of the five samples (C-0, C-2, C-4, F-2, F-4), resulting in 20 genera altogether (Supplementary Table S5). Although it was a small portion compared to the entire community, the core community constituted 55.6% of the total sequences. Overall, *Sphingomonas* (45.7%) and *Methylobacterium* (17.9%) were the most abundant genera (Figure 6).

The correlation analysis between the relative abundance of the 20 genera constituting the core community and the plant performances (Table 2) showed that the relative abundances of *Sphingomonas* and *Salinibacterium* were positively correlated with ST content and plant height at the *r* > 0.8 (*P* < 0.05) level. The genera *Methylobacterium* and *Acinetobacter* were positively correlated with *UGT74G1* and *76G1* gene expression, respectively, while *Agrobacterium* and *Erwinia* were negatively correlated with *UGT74G1* gene expression. Moreover, as growth stage of *Stevia* extended, there were remarkable increases of the relative abundances for positively correlated taxa while the negatively correlated taxa had a sharply decrease. The relative abundance of *Sphingomonas* increased from 7.2 to 47.3% during 4 months of growth which was 5.6 times greater compared to the seedling stage. On the other hand, the relative abundance of *Bacillus* (negatively correlated taxa) decreased from 1.44 to 0.01% during 4 months of growth. Interestingly, FA treatment could strengthen the trend of these changes. At the flowering stage, the relative abundance of *Sphingomonas* was up to 54.4% in F-4 which increased by 15.0% compared to C-4 sample, while the potential plant pathogens (*Erwinia* and *Agrobacterium*) had a clearly decrease.

**Table 2:** **Correlation analysis of the core community and steviol glycoside content**.

**Genus**	**C-0**	**C-2**	**C-4**	**F-2**	**F-4**	**Plant height**	**Content of**			**Gene expression**		
							**ST**	**RC**	**SGs**	**76G1**	**85C2**	**74G1**
	**Relative abundance (%)**					Pearson correlation coefficients^*^						
*Sphingomonas*	4.28	19.2	46.6	16.2	58.8	0.926	0.970					
*Methylobacterium*	4.74	16.5	9.89	20.3	6.92							0.917
*Salinibacterium*	2.48	2.39	3.34	1.16	4.02	0.883	0.925					
*Escherichia*	0.05	1.06	0.19	7.28	0.77						0.883	
*Agrobacterium*	12.4	0.33	1.32	0.12	0.30							−0.914
*Acinetobacter*	3.02	0.97	0.26	0.74	1.92					0.882		
*Erwinia*	7.28	0.63	2.00	0.18	0.19							−0.981
*Hymenobacter*	0.00	1.86	0.28	0.83	1.51							
*Nocardioides*	0.54	2.65	0.07	1.23	0.29							
*Kineococcus*	0.08	1.83	0.21	1.36	0.43							
*Geodermatophilus*	0.16	1.18	0.35	0.55	1.12							
*Serratia*	0.03	0.66	0.25	3.03	0.29							
*Novosphingobium*	3.57	0.08	0.41	0.02	0.96						0.968	
*Stenotrophomonas*	1.09	0.2	0.2	2.03	0.03							
*Pseudomonas*	1.17	0.29	0.31	0.82	0.09						0.907	
*Exiguobacterium*	0	1.59	0	0.16	0							
*Bacillus*	1.44	0.33	0.01	0.39	0.02	−0.990	−0.949	−0.960	−0.884			
*Spirosoma*	0	0.06	0.07	0	0.57							
*Streptococcus*	0	0.11	0.04	0.91	0.07						0.893	
*Rhizobium*	2.13	0	0.06	0	0							

* *Only significant (P < 0.05) correlations with a Pearson correlation coefficient >0.600 or <−0.600 for the dependent variables are shown*.

## Discussion

The present study is the first investigation on the diversity and community composition of endophytic bacteria associated with *S. Rebaudiana*. Comparing with the previous study on leaf endophytes of tomato (Romero et al., 2014), the diversity of the endophytic bacteria in *Stevia* leaves was greater, since the rarefaction curve was saturated at 80 OTUs for endophytic bacteria of tomato, while 595–825 OTUs were detected in the *Stevia* endophytes. This great number of OTUs revealed in the present study also was a little surprise considering the low abundance of endophytic bacteria in leaves (10^3^–10^5^ CFU g^−1^ of fresh leaf for rice, Ferrando et al., 2012) or the low frequencies of isolation 1.6–13.6% for *Trichilia elegans* (Rhoden et al., 2015). The great diversity of endophytic bacteria implied that the leaves of *Stevia* may present a habitat more adequate for the bacteria than those of other plants.

This study revealed that the endophytic bacteria in the *Stevia* leaves were super-dominated by Proteobacteria, followed by Actinobacteria, Bacteroides, Firmicutes, Gemmatemonadetes, and Acidobacteria etc. (Figures 1, 2). This community structure was typical for the phyllosphere of tomato, soybean, clover, *Arabidopsis thaliana*, and *Citrus sinesis* (Yang et al., 2001; Delmotte et al., 2009; Vorholt, 2012; Bodenhausen et al., 2013; Romero et al., 2014), suggesting a common overlay in the key community members at phylum level across host plants. However, the bacterial communities in *Stevia* leaves showed its own specificity compared to other host plants. Gammaproteobacteria was the most abundant class in both the potato and *Arabidopsis thaliana* plants (Berg et al., 2005; Bodenhausen et al., 2013), but Alphaproteobacteria was the most abundant class in the *S. rebaudiana* leaves. Furthermore, the most abundant genera in *Stevia* leaves were *Sphingomonas, Methylobacterium*, and *Acinetobacter* etc. (Supplementary Figure S2); but *Massilia* and *Flavobacterium* were prevalent in *Arabidopsis thaliana* (Bodenhausen et al., 2013) and *Bacillus, Stenotrophomonas*, and *Acinetobacter* etc. were dominant in tomato leaves (Romero et al., 2014). The heavily populated taxon of Candidate division TM7 from tree leaves (Redford et al., 2010) was <0.5% in our samples; and no sequences corresponding to *Pantoea* was observed, which was abundant in the lettuce phyllosphere (Rastogi et al., 2012). On the other hand, the dominance of Enterobacteriaceae in seedling *Stevia* leaves was similar to that of the spinach phyllosphere (Lopez-Velasco et al., 2011) but was different from that in *Arabidopsis thaliana* (Bodenhausen et al., 2013). These variations between host species could be ascribed to the differences in plant characteristics (morphology, physiology, metabolic profile) (Whipps, 2001; Lopez-Velasco et al., 2011). Although some conditions were common for the phyllosphere, such as exposure to UV light, temperature fluctuations, and low nutrient availability (Delmotte et al., 2009), the accumulation of SGs made the *Stevia* leaves more selective for the endophytes, since SGs could be used by some bacteria (Kunova et al., 2014) and inhibit other bacteria (Gamboa and Chaves, 2012). Therefore, our results support the previous estimation that the host species strongly shaped the leaf endophytic community (Ding et al., 2013). As the first study about endophytic bacterial community in *Stevia* leaves, variations in the taxon composition and the proportions of the dominant taxa were observed due to the changes of growth stages. The dominance of Enterobacteriaceae and Rhizobiaceae in seedling leaves and Methylobacteriaceae and Sphingomonadaceae in mature leaves showed a highly dynamic influence on endophytic bacterial communities by plant growth stages. The increase of species richness and decrease of Shannon index in mature leaves (Table 1) also confirmed the great effects of growth stages. These changes might demonstrate that more bacterial species have been harbored, but the dominance of some taxa (*Sphingomonas, Methylobacterium*) was very high in the bacterial community of flowering plants (Table 2). Moreover, the results of principal coordinate analysis (Figure 4) also proved the huge influences of growth stages on the endophytic community. Previously, the variation of endophytic communities related to the plant growth stages have been explicated by temporal changes in abiotic conditions such as temperature, sun exposure, and soil fertilization (Wang et al., 2008a). Moreover, the changes in biotic factors during the plant growth, such as capability of nutrients, leaf size, and glycosides content may also cause the alternation of bacterial endophytes, since the colonization space of endophytes trended to increase as *Stevia* leaf expanding with longer growth time.

Previously, it has been estimated that some of the endophytic bacteria, such as *Sphingomonas* (Innerebner et al., 2011) and *Methylobacterium* (Ardanov et al., 2012), might have potential benefits on plant growth and health. Interestingly, both *Sphingomonas* and *Methylobacterium* were detected as increased and predominant groups in the leaf endophytic community along the growth of *Stevia*, which means they may contribute to the host some kind of benefits. Moreover, a clear decline of highly potential plant pathogens such as *Erwinia* (Toth et al., 2003) and *Agrobacterium* (Pitzschke and Hirt, 2010) was observed, indicating that the plant-endophyte communication trends to form a better relationship for plant health as *Stevia* grew stronger. This study is also the first one to report the regulation of leaf endophytic community structure by fulvic acid. As a plant growth regulator, FA treatment could accelerate the enrichment of beneficial bacteria and decline the potential phytopathogens (Table 2, Figure 4). The decrease of diversity index in the FA treatments (Table 1) was a result of enhanced predominance of some taxa by the application of fulvic acid, mainly the beneficial bacteria *Sphingomonas* and *Salinibacterium* etc. (Table 2). The decrease of *Erwinia* and *Agrobacterium* richness implied that FA treatment may decrease the infection of fire blight diseases and crown gall/hairy root diseases caused by these bacteria (Toth et al., 2003; Pitzschke and Hirt, 2010). This regulation by FA treatment might contribute to the establishment of a harmonious relationship between *Stevia* leaf and endophytic community, and then improve the yield and quality of *Stevia* leaves, such as enhancing the high sweetness and good taste RA content (Table 1).

The CCA results revealed strong correlation between some biotic/abiotic factors, (such as FA treatment and SGs contents) and the endophytic communities. These correlations further confirmed that both the plant (biotic) factors and the abiotic environmental factors could regulate the endophytic microbial community, and in turn the variations of plant endophytes might alter the plant performance as revealed in previous studies on other plants such as *Potato* and *Arabidopsis thaliana* (Berg et al., 2005; Manter et al., 2010; Fernandes et al., 2012; Bodenhausen et al., 2013). The real meaning of the interactions among the FA treatment, the alternation of endophytic bacterial communities, and the plant growth/accumulation of SGs in leaves is still unclear and needs further research. To explain the effects of plant growth and FA treatment on the diversity of endophytic bacteria, the following aspects may be considered: the possible effects of some FA compounds or the metabolites of endophytes induced by the FA on the infection pathways and the establishment of mutualistic relationship (Azevedo et al., 2000); the effects of FA on the chemical and physical states of the wounds or stomata of leaves (Gough et al., 1997; Redford et al., 2010), as well as the stimulation for growth of some plant bacteria, similar to the root exudates for *Bacillus subtilis* (Bais et al., 2006; Rudrappa et al., 2007). The mutualistic relationship between endophytes and plant host suggested a promising potential system for promoting plant performance.

Conclusively, this study provided a general description of the diversity and community shift in the endophyte populations in *Stevia* leaves along the growth stages with/without plant regulator application for the first time. A huge phylogenetic diversity was observed through pyrosequencing technology. The genera *Sphingomonas* and *Methylobacterium* were found as the principal components of the core endophytic community in *Stevia* leaves and presented positive correlations with the stevioside content and *UGT74G1* gene expression. The *Stevia* growth stages could alter the endophytic bacterial community in the *Stevia* leaves, and the FA treatment could accelerate these variations, without changing the pattern of variations along growth period. A significant correlation between some certain species with specific biotic/abiotic factors was also demonstrated. These results demonstrated that the endophytes of *Stevia* leaves might represent a valuable resource for plant growth and steviol glycosides accumulation, and regulation of certain specific plant endophytes to promote plant growth and health might be a reliable option. Further studies on the interactions between the endophytes and plant host should be implemented.

### Conflict of interest statement

The authors declare that the research was conducted in the absence of any commercial or financial relationships that could be construed as a potential conflict of interest.

## Supplementary material

Supplementary Figure S1 **The biosynthetic pathway of steviol glycosides**.

Supplementary Figure S2 **Heatmap of taxonomic distribution in ***Stevia*** leaf samples at different growth stages at the genus level**.

Supplementary Figure S3 **Distribution of the shared and unique OTUs in ***Stevia*** leaf samples at different growth stages (C-0; C-2; C-4)**. Numbers of sequenced reads were presented in parenthesis.

Supplementary Figure S4 **The expression of ***UGT*** genes relative to β-actin in both control group and FA treated leaves at different growth stages**. Mean gene expression followed by an asterisk (^*^) is significant relative to control groups and normalized to β-actin gene (*P* < 0.05). Error bars represent SEM.

Supplementary Table S1 **Community structure of leaf endophytic bacteria at phylum level**.

Supplementary Table S2 **Community structure of leaf endophytic bacteria at family level**.

Supplementary Table S3 **Community structure of leaf endophytic bacteria at genus level**.

Supplementary Table S4 **Distribution of the shared and unique OTUs (at phylum level) in ***Stevia*** leaf samples at different growth stages**.

Supplementary Table S5 **Distribution of the core biome of endophytic bacteria (at genus level) in ***Stevia*** leaf samples at different growth stages**.
