# Supplementary figures and images for: Effects of growth stage and fulvic acid on the diversity and dynamics of endophytic bacterial community in Stevia rebaudiana Bertoni leaves
Source: Front Microbiol. 2015 Aug 25;6:867. doi: 10.3389/fmicb.2015.00867 (PMC4548236; doi:10.3389/fmicb.2015.00867)

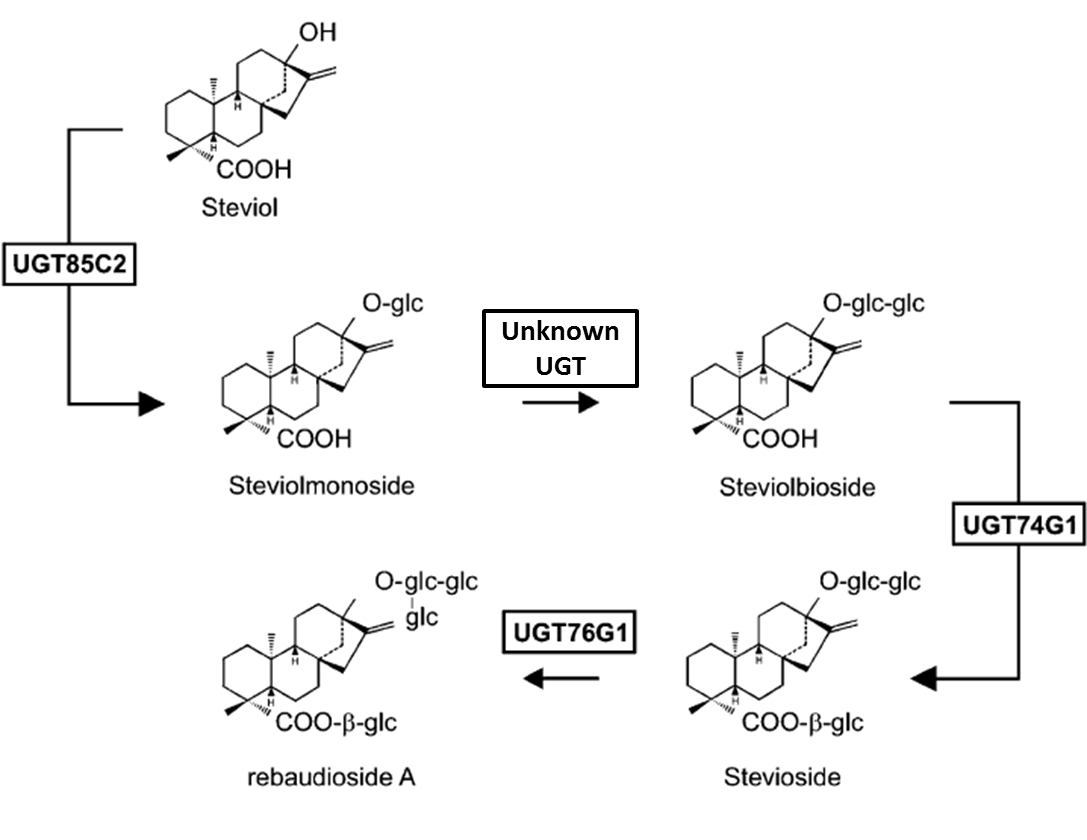

Supplement: Supplementary Figure S1 — The biosynthetic pathway of steviol glycosides. [file Image1.TIF]

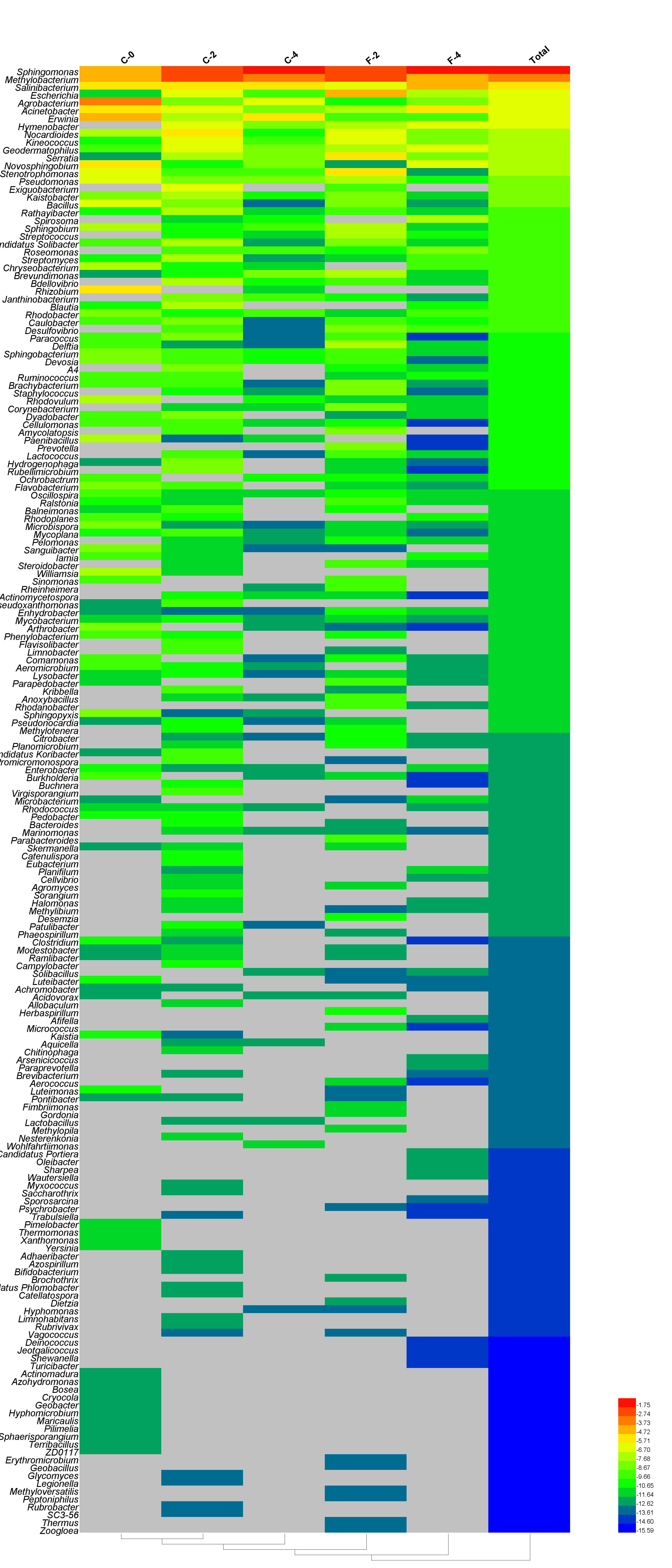

Supplement: Supplementary Figure S2 — Heatmap of taxonomic distribution in Stevia leaf samples at different growth stages at the genus level. [file Image2.TIFF]

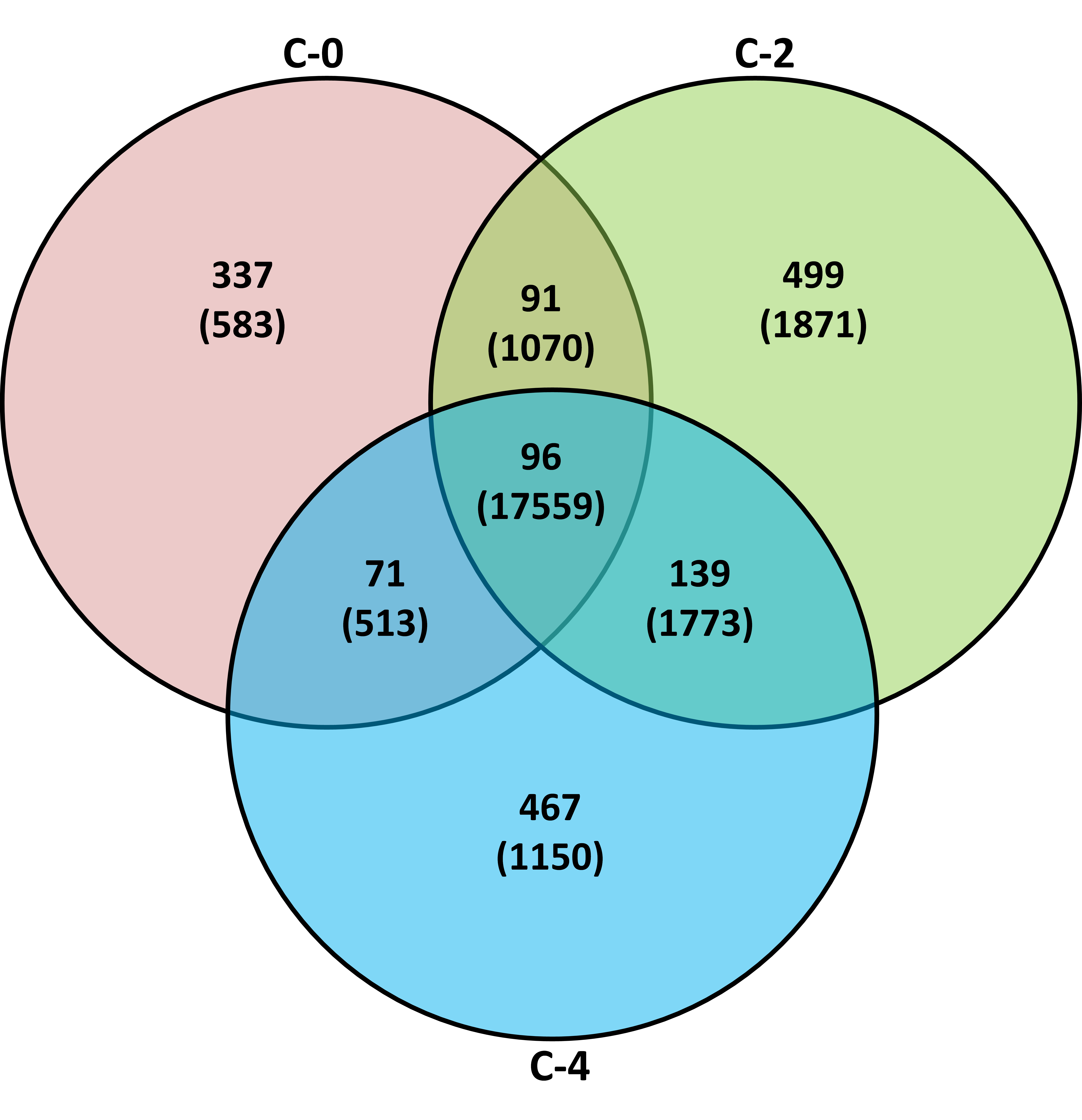

Supplement: Supplementary Figure S3 — Distribution of the shared and unique OTUs in Stevia leaf samples at different growth stages (C-0; C-2; C-4). Numbers of sequenced reads were presented in parenthesis. [file Image3.TIF]
