# Supplementary material for: Effects of growth stage and fulvic acid on the diversity and dynamics of endophytic bacterial community in Stevia rebaudiana Bertoni leaves
Source: Front Microbiol. 2015 Aug 25;6:867. doi: 10.3389/fmicb.2015.00867 (PMC4548236; doi:10.3389/fmicb.2015.00867)
Supplement: Supplementary Table S1 — Community structure of leaf endophytic bacteria at phylum level. [file DataSheet1.DOCX]

**Supplementary Table S1.** Community structure of leaf endophytic bacteria at phylum level

1. Proteobacteria; 2. Actinobacteria; 3. Bacteroidetes; 4. Firmicutes; 5. Gemmatimonadetes; 6. Acidobacteria; 7. Candidate division TM7; 8. Planctomycetes; 9. Armatimonadetes; 10. Chlorobi; 11. Chloroflexi; 12. Termi; 13. Unidentified

| Treatment | Count for | Phylum | | | | | | | | | | | |  | Total |
| --- | --- | --- | --- | --- | --- | --- | --- | --- | --- | --- | --- | --- | --- | --- | --- |
|  |  | 1 | 2 | 3 | 4 | 5 | 6 | 7 | 8 | 9 | 10 | 11 | 12 | 13 |  |
| C-0 | Reads | 2610 | 773 | 87 | 153 | 6 | 15 | 3 | 1 | 0 | 2 | 2 | 0 | 18 | 3670 |
|  | % | **71.12** | **21.06** | **2.37** | **4.17** | 0.16 | 0.41 | 0.08 | 0.03 | 0.00 | 0.05 | 0.05 | 0.00 | 0.49 | 100 |
|  | OTUs | 384 | 128 | 26 | 35 | 6 | 11 | 2 | 1 | 0 | 1 | 1 | 0 | 10 | 605 |
|  | % | **63.47** | **21.16** | **4.30** | **5.79** | 0.99 | 1.82 | 0.33 | 0.17 | 0.00 | 0.17 | 0.17 | 0.00 | 1.65 | 100 |
| C-2 | Reads | 6622 | 2383 | 578 | 405 | 100 | 81 | 3 | 1 | 0 | 0 | 0 | 0 | 55 | 10228 |
|  | % | 64.74 | 23.30 | 5.65 | 3.96 | 0.98 | 0.79 | 0.03 | 0.01 | 0.00 | 0.00 | 0.00 | 0.00 | 0.54 | 100 |
|  | OTUs | 446 | 198 | 98 | 48 | 21 | 12 | 1 | 1 | 0 | 0 | 0 | 0 | 42 | 867 |
|  | % | 51.44 | 22.84 | 11.30 | 5.54 | 2.42 | 1.38 | 0.12 | 0.12 | 0.00 | 0.00 | 0.00 | 0.00 | 4.84 | 100 |
| C-4 | Reads | 9664 | 858 | 105 | 37 | 5 | 4 | 0 | 0 | 0 | 0 | 0 | 0 | 79 | 10752 |
|  | % | 89.88 | 7.98 | 0.98 | 0.34 | 0.05 | 0.04 | 0.00 | 0.00 | 0.00 | 0.00 | 0.00 | 0.00 | 0.73 | 100 |
|  | OTUs | 613 | 92 | 38 | 21 | 5 | 4 | 0 | 0 | 0 |  | 0 | 0 | 37 | 810 |
|  | % | 75.68 | 11.36 | 4.69 | 2.59 | 0.62 | 0.49 | 0.00 | 0.00 | 0.00 | 0.00 | 0.00 | 0.00 | 4.57 | 100 |
| F-2 | Reads | 6358 | 1227 | 169 | 248 | 9 | 23 | 1 | 0 | 4 | 1 | 2 | 1 | 167 | 8210 |
|  | % | 77.44 | 14.95 | 2.06 | 3.02 | 0.11 | 0.28 | 0.01 | 0.00 | 0.05 | 0.01 | 0.02 | 0.01 | 2.03 | 100 |
|  | OTUs | 413 | 131 | 33 | 42 | 2 | 3 | 1 | 0 | 1 | 1 | 1 | 1 | 49 | 678 |
|  | % | 60.91 | 19.32 | 4.87 | 6.19 | 0.29 | 0.44 | 0.15 | 0.00 | 0.15 | 0.15 | 0.15 | 0.15 | 7.23 | 100 |
| F-4 | Reads | 13842 | 1818 | 585 | 113 | 0 | 25 | 0 | 0 | 0 | 0 | 0 | 1 | 45 | 16429 |
|  | % | 84.25 | 11.07 | 3.56 | 0.69 | 0.00 | 0.15 | 0.00 | 0.00 | 0.00 | 0.00 | 0.00 | 0.01 | 0.27 | 100 |
|  | OTUs | 432 | 98 | 62 | 33 | 0 | 6 | 0 | 0 | 0 | 0 | 0 | 1 | 32 | 664 |
|  | % | 65.06 | 14.76 | 9.34 | 4.97 | 0.00 | 0.90 | 0.00 | 0.00 | 0.00 | 0.00 | 0.00 | 0.15 | 4.82 | 100 |
| Total | Reads | 39096 | 7059 | 1524 | 956 | 120 | 148 | 7 | 2 | 4 | 3 | 4 | 2 | 364 | 49289 |
|  | % | 79.32 | 14.32 | 3.09 | 1.94 | 0.24 | 0.30 | 0.01 | 0.00 | 0.01 | 0.01 | 0.01 | 0.00 | 0.74 | 100 |
|  | OTUs | 1257 | 413 | 195 | 124 | 30 | 29 | 4 | 2 | 1 | 2 | 2 | 2 | 103 | 2164 |
|  | % | 58.09 | 19.09 | 9.01 | 5.73 | 1.39 | 1.34 | 0.18 | 0.09 | 0.05 | 0.09 | 0.09 | 0.09 | 4.76 | 100 |

Among the 12 phyla, Proteobacteria was the most abundant and divergent group in all the five samples, presenting 65.09 %-90.55 % (79.91 % in total) of the reads and 54.06 %-79.30 % (60.99 % in total) of the OTUs. The secondary abundant and divergent phylum was Actinobacteria, presenting 8.04 %-23.41 % (14.43 in total) of the reads and 11.90 %-24.00 % (20.04 % in total) of the OTUs. The phyla Bacteroidetes and Firmicutes presented more than 1 % of reads in four of the samples (except C-4) and 2.72 %-11.88 % of OTUs. The other eight phyla presented less than 1 % of the reads and the OTUs in most cases, and only the phyla Gemmatimonadetes and Acidobacteria occupied 1.01 %-2.25 % of OTUs in C-0 and C-2.

**Supplementary Table S2.** Community structure of leaf endophytic bacteria at Family level

| Family | CK-0 | | | | CK-2 | | | | CK-4 | | | | F-2 | | | | F-4 | | | | Total | | | |  |
| --- | --- | --- | --- | --- | --- | --- | --- | --- | --- | --- | --- | --- | --- | --- | --- | --- | --- | --- | --- | --- | --- | --- | --- | --- | --- |
|  | Reads | % | OTU | % | Reads | % | OTU | % | Reads | % | OTU | % | Reads | % | OTU | % | Reads | % | OTU | % | Reads | % | OTU | % | |
| Sphingomonadaceae | 361 | 10.52 | 50 | 10.92 | 2124 | 22.67 | 81 | 12.98 | 5394 | 53.20 | 152 | 27.69 | 1445 | 19.06 | 65 | 13.24 | 10041 | 62.59 | 127 | 25.25 | 19365 | 41.59 | 236 | 15.26 |  |
| Methylobacteriaceae | 206 | 6.01 | 18 | 3.93 | 2593 | 27.68 | 55 | 8.81 | 1829 | 18.04 | 81 | 14.75 | 2509 | 33.09 | 69 | 14.05 | 1797 | 11.20 | 49 | 9.74 | 8934 | 19.19 | 124 | 8.02 |  |
| Enterobacteriaceae | 632 | 18.43 | 53 | 11.57 | 419 | 4.47 | 33 | 5.29 | 434 | 4.28 | 45 | 8.20 | 947 | 12.49 | 24 | 4.89 | 606 | 3.78 | 43 | 8.55 | 3038 | 6.52 | 102 | 6.59 |  |
| Microbacteriaceae | 190 | 5.54 | 16 | 3.49 | 499 | 5.33 | 18 | 2.88 | 537 | 5.30 | 26 | 4.74 | 169 | 2.23 | 12 | 2.44 | 789 | 4.92 | 17 | 3.38 | 2184 | 4.69 | 51 | 3.30 |  |
| Kineosporiaceae | 3 | 0.09 | 1 | 0.22 | 414 | 4.42 | 8 | 1.28 | 162 | 1.60 | 8 | 1.46 | 369 | 4.87 | 8 | 1.63 | 571 | 3.56 | 9 | 1.79 | 1519 | 3.26 | 15 | 0.97 |  |
| Pseudomonadaceae | 198 | 5.77 | 23 | 5.02 | 205 | 2.19 | 14 | 2.24 | 463 | 4.57 | 22 | 4.01 | 235 | 3.10 | 23 | 4.68 | 102 | 0.64 | 13 | 2.58 | 1203 | 2.58 | 48 | 3.10 |  |
| Aurantimonadaceae | 9 | 0.26 | 4 | 0.87 | 114 | 1.22 | 5 | 0.80 | 573 | 5.65 | 14 | 2.55 | 91 | 1.20 | 4 | 0.81 | 355 | 2.21 | 10 | 1.99 | 1142 | 2.45 | 15 | 0.97 |  |
| Nocardioidaceae | 55 | 1.60 | 21 | 4.59 | 536 | 5.72 | 29 | 4.65 | 14 | 0.14 | 7 | 1.28 | 216 | 2.85 | 21 | 4.28 | 94 | 0.59 | 13 | 2.58 | 915 | 1.97 | 53 | 3.43 |  |
| Rhizobiaceae | 599 | 17.46 | 30 | 6.55 | 49 | 0.52 | 8 | 1.28 | 165 | 1.63 | 11 | 2.00 | 13 | 0.17 | 2 | 0.41 | 56 | 0.35 | 4 | 0.80 | 882 | 1.89 | 33 | 2.13 |  |
| Flexibacteraceae | 6 | 0.17 | 4 | 0.87 | 241 | 2.57 | 29 | 4.65 | 40 | 0.39 | 11 | 2.00 | 74 | 0.98 | 15 | 3.05 | 364 | 2.27 | 14 | 2.78 | 725 | 1.56 | 45 | 2.91 |  |
| Moraxellaceae | 112 | 3.27 | 12 | 2.62 | 101 | 1.08 | 12 | 1.92 | 29 | 0.29 | 6 | 1.09 | 70 | 0.92 | 14 | 2.85 | 326 | 2.03 | 16 | 3.18 | 638 | 1.37 | 36 | 2.33 |  |
| Xanthomonadaceae | 170 | 4.96 | 22 | 4.80 | 68 | 0.73 | 11 | 1.76 | 41 | 0.40 | 12 | 2.19 | 211 | 2.78 | 15 | 3.05 | 31 | 0.19 | 11 | 2.19 | 521 | 1.12 | 53 | 3.43 |  |
| Micrococcaceae | 306 | 8.92 | 13 | 2.84 | 90 | 0.96 | 6 | 0.96 | 7 | 0.07 | 4 | 0.73 | 81 | 1.07 | 7 | 1.43 | 13 | 0.08 | 6 | 1.19 | 497 | 1.07 | 21 | 1.36 |  |
| Geodermatophilaceae | 10 | 0.29 | 4 | 0.87 | 162 | 1.73 | 8 | 1.28 | 39 | 0.38 | 4 | 0.73 | 68 | 0.90 | 6 | 1.22 | 187 | 1.17 | 4 | 0.80 | 466 | 1.00 | 14 | 0.90 |  |
| Caulobacteraceae | 28 | 0.82 | 7 | 1.53 | 92 | 0.98 | 12 | 1.92 | 54 | 0.53 | 14 | 2.55 | 81 | 1.07 | 10 | 2.04 | 49 | 0.31 | 9 | 1.79 | 304 | 0.65 | 28 | 1.81 |  |
| Comamonadaceae | 22 | 0.64 | 12 | 2.62 | 84 | 0.90 | 20 | 3.21 | 63 | 0.62 | 17 | 3.10 | 77 | 1.02 | 13 | 2.65 | 56 | 0.35 | 15 | 2.98 | 302 | 0.65 | 47 | 3.04 |  |
| Oxalobacteraceae | 10 | 0.29 | 4 | 0.87 | 99 | 1.06 | 21 | 3.37 | 38 | 0.37 | 8 | 1.46 | 93 | 1.23 | 11 | 2.24 | 15 | 0.09 | 4 | 0.80 | 255 | 0.55 | 30 | 1.94 |  |
| Rhodobacteraceae | 59 | 1.72 | 17 | 3.71 | 84 | 0.90 | 12 | 1.92 | 36 | 0.36 | 7 | 1.28 | 40 | 0.53 | 14 | 2.85 | 35 | 0.22 | 6 | 1.19 | 254 | 0.55 | 35 | 2.26 |  |
| Sphingobacteriaceae | 32 | 0.93 | 10 | 2.18 | 47 | 0.50 | 12 | 1.92 | 23 | 0.23 | 12 | 2.19 | 28 | 0.37 | 5 | 1.02 | 53 | 0.33 | 10 | 1.99 | 183 | 0.39 | 32 | 2.07 |  |
| Exiguobacteraceae | 0 | 0.00 | 0 | 0.00 | 164 | 1.75 | 8 | 1.28 | 0 | 0.00 | 0 | 0.00 | 13 | 0.17 | 4 | 0.81 | 0 | 0.00 | 0 | 0.00 | 177 | 0.38 | 8 | 0.52 |  |
| Flavobacteriaceae | 45 | 1.31 | 9 | 1.97 | 26 | 0.28 | 9 | 1.44 | 21 | 0.21 | 5 | 0.91 | 8 | 0.11 | 5 | 1.02 | 64 | 0.40 | 12 | 2.39 | 164 | 0.35 | 33 | 2.13 |  |
| Bacillaceae | 55 | 1.60 | 6 | 1.31 | 38 | 0.41 | 6 | 0.96 | 4 | 0.04 | 3 | 0.55 | 45 | 0.59 | 8 | 1.63 | 3 | 0.02 | 1 | 0.20 | 145 | 0.31 | 13 | 0.84 |  |
| Micromonosporaceae | 7 | 0.20 | 5 | 1.09 | 108 | 1.15 | 13 | 2.08 | 2 | 0.02 | 2 | 0.36 | 9 | 0.12 | 4 | 0.81 | 15 | 0.09 | 3 | 0.60 | 141 | 0.30 | 17 | 1.10 |  |
| Streptococcaceae | 0 | 0.00 | 0 | 0.00 | 24 | 0.26 | 4 | 0.64 | 5 | 0.05 | 4 | 0.73 | 90 | 1.19 | 7 | 1.43 | 19 | 0.12 | 6 | 1.19 | 138 | 0.30 | 9 | 0.58 |  |
| Streptomycetaceae | 7 | 0.20 | 2 | 0.44 | 62 | 0.66 | 6 | 0.96 | 2 | 0.02 | 1 | 0.18 | 5 | 0.07 | 1 | 0.20 | 42 | 0.26 | 6 | 1.19 | 118 | 0.25 | 10 | 0.65 |  |
| Acetobacteraceae | 2 | 0.06 | 1 | 0.22 | 18 | 0.19 | 5 | 0.80 | 22 | 0.22 | 5 | 0.91 | 9 | 0.12 | 3 | 0.61 | 60 | 0.37 | 4 | 0.80 | 111 | 0.24 | 9 | 0.58 |  |
| Lachnospiraceae | 4 | 0.12 | 2 | 0.44 | 66 | 0.70 | 6 | 0.96 | 1 | 0.01 | 1 | 0.18 | 4 | 0.05 | 2 | 0.41 | 34 | 0.21 | 5 | 0.99 | 109 | 0.23 | 13 | 0.84 |  |
| Pseudonocardiaceae | 1 | 0.03 | 1 | 0.22 | 44 | 0.47 | 8 | 1.28 | 7 | 0.07 | 4 | 0.73 | 51 | 0.67 | 8 | 1.63 | 1 | 0.01 | 1 | 0.20 | 104 | 0.22 | 16 | 1.03 |  |
| Intrasporangiaceae | 29 | 0.85 | 2 | 0.44 | 52 | 0.56 | 2 | 0.32 | 0 | 0.00 | 0 | 0.00 | 13 | 0.17 | 1 | 0.20 | 9 | 0.06 | 2 | 0.40 | 103 | 0.22 | 3 | 0.19 |  |
| Solibacteraceae | 6 | 0.17 | 4 | 0.87 | 55 | 0.59 | 7 | 1.12 | 3 | 0.03 | 3 | 0.55 | 22 | 0.29 | 2 | 0.41 | 8 | 0.05 | 1 | 0.20 | 94 | 0.20 | 13 | 0.84 |  |
| Hyphomicrobiaceae | 22 | 0.64 | 12 | 2.62 | 29 | 0.31 | 8 | 1.28 | 7 | 0.07 | 2 | 0.36 | 15 | 0.20 | 5 | 1.02 | 20 | 0.12 | 5 | 0.99 | 93 | 0.20 | 18 | 1.16 |  |
| Bdellovibrionaceae | 0 | 0.00 | 0 | 0.00 | 53 | 0.57 | 1 | 0.16 | 9 | 0.09 | 2 | 0.36 | 18 | 0.24 | 3 | 0.61 | 6 | 0.04 | 1 | 0.20 | 86 | 0.18 | 4 | 0.26 |  |
| Chitinophagaceae | 0 | 0.00 | 0 | 0.00 | 66 | 0.70 | 18 | 2.88 | 3 | 0.03 | 2 | 0.36 | 1 | 0.01 | 1 | 0.20 | 14 | 0.09 | 8 | 1.59 | 84 | 0.18 | 26 | 1.68 |  |
| Ruminococcaceae | 13 | 0.38 | 8 | 1.75 | 21 | 0.22 | 3 | 0.48 | 9 | 0.09 | 2 | 0.36 | 16 | 0.21 | 4 | 0.81 | 22 | 0.14 | 5 | 0.99 | 81 | 0.17 | 21 | 1.36 |  |
| Paenibacillaceae | 58 | 1.69 | 11 | 2.40 | 8 | 0.09 | 2 | 0.32 | 6 | 0.06 | 2 | 0.36 | 0 | 0.00 | 0 | 0.00 | 1 | 0.01 | 1 | 0.20 | 73 | 0.16 | 14 | 0.90 |  |
| Bradyrhizobiaceae | 8 | 0.23 | 5 | 1.09 | 33 | 0.35 | 3 | 0.48 | 2 | 0.02 | 1 | 0.18 | 24 | 0.32 | 3 | 0.61 | 5 | 0.03 | 2 | 0.40 | 72 | 0.15 | 7 | 0.45 |  |
| Desulfovibrionaceae | 0 | 0.00 | 0 | 0.00 | 15 | 0.16 | 3 | 0.48 | 1 | 0.01 | 1 | 0.18 | 24 | 0.32 | 1 | 0.20 | 23 | 0.14 | 2 | 0.40 | 63 | 0.14 | 3 | 0.19 |  |
| Gaiellaceae | 12 | 0.35 | 7 | 1.53 | 13 | 0.14 | 8 | 1.28 | 0 | 0.00 | 0 | 0.00 | 36 | 0.47 | 5 | 1.02 | 0 | 0.00 | 0 | 0.00 | 61 | 0.13 | 14 | 0.90 |  |
| Sinobacteraceae | 1 | 0.03 | 1 | 0.22 | 15 | 0.16 | 9 | 1.44 | 7 | 0.07 | 2 | 0.36 | 19 | 0.25 | 5 | 1.02 | 12 | 0.07 | 2 | 0.40 | 54 | 0.12 | 15 | 0.97 |  |
| Flammeovirgaceae | 0 | 0.00 | 0 | 0.00 | 37 | 0.39 | 9 | 1.44 | 0 | 0.00 | 0 | 0.00 | 6 | 0.08 | 1 | 0.20 | 9 | 0.06 | 1 | 0.20 | 52 | 0.11 | 11 | 0.71 |  |
| Dermabacteraceae | 10 | 0.29 | 2 | 0.44 | 13 | 0.14 | 1 | 0.16 | 1 | 0.01 | 1 | 0.18 | 23 | 0.30 | 3 | 0.61 | 4 | 0.02 | 1 | 0.20 | 51 | 0.11 | 5 | 0.32 |  |
| Cellulomonadaceae | 6 | 0.17 | 1 | 0.22 | 29 | 0.31 | 2 | 0.32 | 4 | 0.04 | 1 | 0.18 | 9 | 0.12 | 1 | 0.20 | 1 | 0.01 | 1 | 0.20 | 49 | 0.11 | 2 | 0.13 |  |
| Rhodospirillaceae | 6 | 0.17 | 5 | 1.09 | 24 | 0.26 | 8 | 1.28 | 4 | 0.04 | 3 | 0.55 | 9 | 0.12 | 5 | 1.02 | 6 | 0.04 | 2 | 0.40 | 49 | 0.11 | 18 | 1.16 |  |
| Staphylococcaceae | 0 | 0.00 | 0 | 0.00 | 11 | 0.12 | 2 | 0.32 | 2 | 0.02 | 2 | 0.36 | 31 | 0.41 | 4 | 0.81 | 3 | 0.02 | 3 | 0.60 | 47 | 0.10 | 6 | 0.39 |  |
| Corynebacteriaceae | 0 | 0.00 | 0 | 0.00 | 6 | 0.06 | 3 | 0.48 | 5 | 0.05 | 2 | 0.36 | 27 | 0.36 | 4 | 0.81 | 6 | 0.04 | 3 | 0.60 | 44 | 0.09 | 7 | 0.45 |  |
| Prevotellaceae | 0 | 0.00 | 0 | 0.00 | 0 | 0.00 | 0 | 0.00 | 0 | 0.00 | 0 | 0.00 | 36 | 0.47 | 1 | 0.20 | 0 | 0.00 | 0 | 0.00 | 36 | 0.08 | 1 | 0.06 |  |
| Koribacteraceae | 2 | 0.06 | 2 | 0.44 | 23 | 0.25 | 4 | 0.64 | 0 | 0.00 | 0 | 0.00 | 0 | 0.00 | 0 | 0.00 | 10 | 0.06 | 3 | 0.60 | 35 | 0.08 | 7 | 0.45 |  |
| Chromatiaceae | 0 | 0.00 | 0 | 0.00 | 0 | 0.00 | 0 | 0.00 | 2 | 0.02 | 1 | 0.18 | 24 | 0.32 | 2 | 0.41 | 7 | 0.04 | 1 | 0.20 | 33 | 0.07 | 3 | 0.19 |  |
| Actinosynnemataceae | 1 | 0.03 | 1 | 0.22 | 30 | 0.32 | 0 | 0.00 | 0 | 0.00 | 0 | 0.00 | 1 | 0.01 | 1 | 0.20 | 0 | 0.00 | 0 | 0.00 | 32 | 0.07 | 3 | 0.19 |  |
| Brucellaceae | 9 | 0.26 | 1 | 0.22 | 0 | 0.00 | 0 | 0.00 | 7 | 0.07 | 1 | 0.18 | 10 | 0.13 | 2 | 0.41 | 6 | 0.04 | 1 | 0.20 | 32 | 0.07 | 2 | 0.13 |  |
| Lactobacillaceae | 0 | 0.00 | 0 | 0.00 | 14 | 0.15 | 3 | 0.48 | 5 | 0.05 | 3 | 0.55 | 12 | 0.16 | 1 | 0.20 | 0 | 0.00 | 0 | 0.00 | 31 | 0.07 | 4 | 0.26 |  |
| Planococcaceae | 2 | 0.06 | 1 | 0.22 | 6 | 0.06 | 2 | 0.32 | 3 | 0.03 | 2 | 0.36 | 10 | 0.13 | 3 | 0.61 | 8 | 0.05 | 3 | 0.60 | 29 | 0.06 | 5 | 0.32 |  |
| Burkholderiaceae | 7 | 0.20 | 5 | 1.09 | 0 | 0.00 | 0 | 0.00 | 3 | 0.03 | 3 | 0.55 | 16 | 0.21 | 2 | 0.41 | 1 | 0.01 | 1 | 0.20 | 27 | 0.06 | 10 | 0.65 |  |
| Erythrobacteraceae | 2 | 0.06 | 1 | 0.22 | 16 | 0.17 | 4 | 0.64 | 5 | 0.05 | 2 | 0.36 | 3 | 0.04 | 3 | 0.61 | 1 | 0.01 | 1 | 0.20 | 27 | 0.06 | 6 | 0.39 |  |
| Haliangiaceae | 0 | 0.00 | 0 | 0.00 | 16 | 0.17 | 4 | 0.64 | 1 | 0.01 | 1 | 0.18 | 6 | 0.08 | 1 | 0.20 | 0 | 0.00 | 0 | 0.00 | 23 | 0.05 | 6 | 0.39 |  |
| Sanguibacteraceae | 16 | 0.47 | 1 | 0.22 | 5 | 0.05 | 1 | 0.16 | 1 | 0.01 | 1 | 0.18 | 1 | 0.01 | 1 | 0.20 | 0 | 0.00 | 0 | 0.00 | 23 | 0.05 | 1 | 0.06 |  |
| Sporichthyaceae | 10 | 0.29 | 1 | 0.22 | 11 | 0.12 | 1 | 0.16 | 0 | 0.00 | 0 | 0.00 | 2 | 0.03 | 1 | 0.20 | 0 | 0.00 | 0 | 0.00 | 23 | 0.05 | 1 | 0.06 |  |
| Iamiaceae | 7 | 0.20 | 2 | 0.44 | 4 | 0.04 | 1 | 0.16 | 0 | 0.00 | 0 | 0.00 | 0 | 0.00 | 0 | 0.00 | 11 | 0.07 | 1 | 0.20 | 22 | 0.05 | 2 | 0.13 |  |
| Williamsiaceae | 18 | 0.52 | 1 | 0.22 | 4 | 0.04 | 2 | 0.32 | 0 | 0.00 | 0 | 0.00 | 0 | 0.00 | 0 | 0.00 | 0 | 0.00 | 0 | 0.00 | 22 | 0.05 | 2 | 0.13 |  |
| Methylophilaceae | 0 | 0.00 | 0 | 0.00 | 15 | 0.16 | 5 | 0.80 | 0 | 0.00 | 0 | 0.00 | 6 | 0.08 | 2 | 0.41 | 0 | 0.00 | 0 | 0.00 | 21 | 0.05 | 7 | 0.45 |  |
| Erysipelotrichaceae | 0 | 0.00 | 0 | 0.00 | 16 | 0.17 | 3 | 0.48 | 0 | 0.00 | 0 | 0.00 | 4 | 0.05 | 1 | 0.20 | 0 | 0.00 | 0 | 0.00 | 20 | 0.04 | 4 | 0.26 |  |
| Mycobacteriaceae | 2 | 0.06 | 2 | 0.44 | 8 | 0.09 | 3 | 0.48 | 2 | 0.02 | 2 | 0.36 | 3 | 0.04 | 2 | 0.41 | 5 | 0.03 | 2 | 0.40 | 20 | 0.04 | 6 | 0.39 |  |
| Streptosporangiaceae | 3 | 0.09 | 2 | 0.44 | 4 | 0.04 | 1 | 0.16 | 0 | 0.00 | 0 | 0.00 | 13 | 0.17 | 2 | 0.41 | 0 | 0.00 | 0 | 0.00 | 20 | 0.04 | 5 | 0.32 |  |
| Alcaligenaceae | 10 | 0.29 | 2 | 0.44 | 3 | 0.03 | 1 | 0.16 | 0 | 0.00 | 0 | 0.00 | 4 | 0.05 | 1 | 0.20 | 2 | 0.01 | 1 | 0.20 | 19 | 0.04 | 3 | 0.19 |  |
| Patulibacteraceae | 1 | 0.03 | 1 | 0.22 | 9 | 0.10 | 2 | 0.32 | 3 | 0.03 | 2 | 0.36 | 6 | 0.08 | 4 | 0.81 | 0 | 0.00 | 0 | 0.00 | 19 | 0.04 | 8 | 0.52 |  |
| Solirubrobacteraceae | 0 | 0.00 | 0 | 0.00 | 10 | 0.11 | 3 | 0.48 | 0 | 0.00 | 0 | 0.00 | 9 | 0.12 | 4 | 0.81 | 0 | 0.00 | 0 | 0.00 | 19 | 0.04 | 5 | 0.32 |  |
| Bacteroidaceae | 0 | 0.00 | 0 | 0.00 | 10 | 0.11 | 2 | 0.32 | 0 | 0.00 | 0 | 0.00 | 2 | 0.03 | 1 | 0.20 | 4 | 0.02 | 2 | 0.40 | 16 | 0.03 | 5 | 0.32 |  |
| Beijerinckiaceae | 2 | 0.06 | 1 | 0.22 | 0 | 0.00 | 1 | 0.16 | 10 | 0.10 | 1 | 0.18 | 0 | 0.00 | 0 | 0.00 | 2 | 0.01 | 1 | 0.20 | 14 | 0.03 | 1 | 0.06 |  |
| Oceanospirillaceae | 0 | 0.00 | 0 | 0.00 | 4 | 0.04 | 1 | 0.16 | 3 | 0.03 | 2 | 0.36 | 2 | 0.03 | 1 | 0.20 | 5 | 0.03 | 2 | 0.40 | 14 | 0.03 | 3 | 0.19 |  |
| Promicromonosporaceae | 0 | 0.00 | 0 | 0.00 | 13 | 0.14 | 2 | 0.32 | 0 | 0.00 | 0 | 0.00 | 1 | 0.01 | 1 | 0.20 | 0 | 0.00 | 0 | 0.00 | 14 | 0.03 | 1 | 0.06 |  |
| Alteromonadaceae | 1 | 0.03 | 1 | 0.22 | 9 | 0.10 | 1 | 0.16 | 0 | 0.00 | 0 | 0.00 | 0 | 0.00 | 0 | 0.00 | 3 | 0.02 | 1 | 0.20 | 13 | 0.03 | 4 | 0.26 |  |
| Saprospiraceae | 2 | 0.06 | 2 | 0.44 | 8 | 0.09 | 1 | 0.16 | 2 | 0.02 | 1 | 0.18 | 1 | 0.01 | 1 | 0.20 | 0 | 0.00 | 0 | 0.00 | 13 | 0.03 | 3 | 0.19 |  |
| Halomonadaceae | 0 | 0.00 | 0 | 0.00 | 4 | 0.04 | 2 | 0.32 | 1 | 0.01 | 1 | 0.18 | 0 | 0.00 | 0 | 0.00 | 7 | 0.04 | 3 | 0.60 | 12 | 0.03 | 5 | 0.32 |  |
| Nocardiaceae | 2 | 0.06 | 1 | 0.22 | 4 | 0.04 | 1 | 0.16 | 2 | 0.02 | 1 | 0.18 | 0 | 0.00 | 0 | 0.00 | 4 | 0.02 | 1 | 0.20 | 12 | 0.03 | 3 | 0.19 |  |
| Porphyromonadaceae | 0 | 0.00 | 0 | 0.00 | 0 | 0.00 | 0 | 0.00 | 0 | 0.00 | 0 | 0.00 | 11 | 0.15 | 2 | 0.41 | 0 | 0.00 | 0 | 0.00 | 11 | 0.02 | 2 | 0.13 |  |
| Catenulisporaceae | 0 | 0.00 | 0 | 0.00 | 10 | 0.11 | 1 | 0.16 | 0 | 0.00 | 0 | 0.00 | 0 | 0.00 | 0 | 0.00 | 0 | 0.00 | 0 | 0.00 | 10 | 0.02 | 1 | 0.06 |  |
| Polyangiaceae | 1 | 0.03 | 1 | 0.22 | 9 | 0.10 | 1 | 0.16 | 0 | 0.00 | 0 | 0.00 | 0 | 0.00 | 0 | 0.00 | 0 | 0.00 | 0 | 0.00 | 10 | 0.02 | 2 | 0.13 |  |
| Clostridiaceae | 3 | 0.09 | 2 | 0.44 | 3 | 0.03 | 1 | 0.16 | 0 | 0.00 | 0 | 0.00 | 2 | 0.03 | 2 | 0.41 | 1 | 0.01 | 1 | 0.20 | 9 | 0.02 | 5 | 0.32 |  |
| Thermoactinomycetaceae | 0 | 0.00 | 0 | 0.00 | 3 | 0.03 | 1 | 0.16 | 0 | 0.00 | 0 | 0.00 | 0 | 0.00 | 0 | 0.00 | 6 | 0.04 | 2 | 0.40 | 9 | 0.02 | 2 | 0.13 |  |
| Carnobacteriaceae | 0 | 0.00 | 0 | 0.00 | 0 | 0.00 | 0 | 0.00 | 0 | 0.00 | 0 | 0.00 | 8 | 0.11 | 1 | 0.20 | 0 | 0.00 | 0 | 0.00 | 8 | 0.02 | 1 | 0.06 |  |
| Coxiellaceae | 0 | 0.00 | 0 | 0.00 | 3 | 0.03 | 2 | 0.32 | 3 | 0.03 | 1 | 0.18 | 2 | 0.03 | 1 | 0.20 | 0 | 0.00 | 0 | 0.00 | 8 | 0.02 | 4 | 0.26 |  |
| Rikenellaceae | 0 | 0.00 | 0 | 0.00 | 8 | 0.09 | 1 | 0.16 | 0 | 0.00 | 0 | 0.00 | 0 | 0.00 | 0 | 0.00 | 0 | 0.00 | 0 | 0.00 | 8 | 0.02 | 1 | 0.06 |  |
| Acidobacteriaceae | 1 | 0.03 | 1 | 0.22 | 3 | 0.03 | 1 | 0.16 | 1 | 0.01 | 1 | 0.18 | 0 | 0.00 | 0 | 0.00 | 2 | 0.01 | 1 | 0.20 | 7 | 0.02 | 3 | 0.19 |  |
| Campylobacteraceae | 0 | 0.00 | 0 | 0.00 | 7 | 0.07 | 1 | 0.16 | 0 | 0.00 | 0 | 0.00 | 0 | 0.00 | 0 | 0.00 | 0 | 0.00 | 0 | 0.00 | 7 | 0.02 | 1 | 0.06 |  |
| Frankiaceae | 4 | 0.12 | 2 | 0.44 | 0 | 0.00 | 0 | 0.00 | 3 | 0.03 | 1 | 0.18 | 0 | 0.00 | 0 | 0.00 | 0 | 0.00 | 0 | 0.00 | 7 | 0.02 | 2 | 0.13 |  |
| Phyllobacteriaceae | 3 | 0.09 | 3 | 0.66 | 2 | 0.02 | 2 | 0.32 | 2 | 0.02 | 2 | 0.36 | 0 | 0.00 | 0 | 0.00 | 0 | 0.00 | 0 | 0.00 | 7 | 0.02 | 5 | 0.32 |  |
| Rhodocyclaceae | 3 | 0.09 | 1 | 0.22 | 1 | 0.01 | 1 | 0.16 | 0 | 0.00 | 0 | 0.00 | 3 | 0.04 | 3 | 0.61 | 0 | 0.00 | 0 | 0.00 | 7 | 0.02 | 5 | 0.32 |  |
| Thermoanaerobacteraceae | 1 | 0.03 | 1 | 0.22 | 0 | 0.00 | 0 | 0.00 | 0 | 0.00 | 0 | 0.00 | 6 | 0.08 | 1 | 0.20 | 0 | 0.00 | 0 | 0.00 | 7 | 0.02 | 2 | 0.13 |  |
| Aerococcaceae | 0 | 0.00 | 0 | 0.00 | 0 | 0.00 | 0 | 0.00 | 0 | 0.00 | 0 | 0.00 | 3 | 0.04 | 1 | 0.20 | 3 | 0.02 | 2 | 0.40 | 6 | 0.01 | 2 | 0.13 |  |
| Cystobacterineae | 0 | 0.00 | 0 | 0.00 | 1 | 0.01 | 1 | 0.16 | 0 | 0.00 | 0 | 0.00 | 3 | 0.04 | 1 | 0.20 | 2 | 0.01 | 1 | 0.20 | 6 | 0.01 | 2 | 0.13 |  |
| Hyphomonadaceae | 1 | 0.03 | 1 | 0.22 | 1 | 0.01 | 1 | 0.16 | 1 | 0.01 | 1 | 0.18 | 2 | 0.03 | 2 | 0.41 | 1 | 0.01 | 1 | 0.20 | 6 | 0.01 | 5 | 0.32 |  |
| Beutenbergiaceae | 3 | 0.09 | 1 | 0.22 | 0 | 0.00 | 1 | 0.16 | 0 | 0.00 | 0 | 0.00 | 0 | 0.00 | 0 | 0.00 | 2 | 0.01 | 1 | 0.20 | 5 | 0.01 | 1 | 0.06 |  |
| Ellin5301 | 2 | 0.06 | 2 | 0.44 | 2 | 0.02 | 0 | 0.00 | 1 | 0.01 | 1 | 0.18 | 0 | 0.00 | 0 | 0.00 | 0 | 0.00 | 0 | 0.00 | 5 | 0.01 | 4 | 0.26 |  |
| Myxococcaceae | 1 | 0.03 | 1 | 0.22 | 3 | 0.03 | 0 | 0.00 | 1 | 0.01 | 1 | 0.18 | 0 | 0.00 | 0 | 0.00 | 0 | 0.00 | 0 | 0.00 | 5 | 0.01 | 3 | 0.19 |  |
| Paraprevotellaceae | 0 | 0.00 | 0 | 0.00 | 0 | 0.00 | 0 | 0.00 | 0 | 0.00 | 0 | 0.00 | 0 | 0.00 | 0 | 0.00 | 5 | 0.03 | 2 | 0.40 | 5 | 0.01 | 2 | 0.13 |  |
| Rhodobiaceae | 0 | 0.00 | 0 | 0.00 | 0 | 0.00 | 0 | 0.00 | 0 | 0.00 | 0 | 0.00 | 0 | 0.00 | 0 | 0.00 | 5 | 0.03 | 1 | 0.20 | 5 | 0.01 | 1 | 0.06 |  |
| Aeromonadaceae | 0 | 0.00 | 0 | 0.00 | 0 | 0.00 | 0 | 0.00 | 0 | 0.00 | 0 | 0.00 | 4 | 0.05 | 1 | 0.20 | 0 | 0.00 | 0 | 0.00 | 4 | 0.01 | 1 | 0.06 |  |
| Brevibacteriaceae | 0 | 0.00 | 0 | 0.00 | 2 | 0.02 | 0 | 0.00 | 0 | 0.00 | 0 | 0.00 | 0 | 0.00 | 0 | 0.00 | 2 | 0.01 | 1 | 0.20 | 4 | 0.01 | 2 | 0.13 |  |
| Fimbriimonadaceae | 0 | 0.00 | 0 | 0.00 | 0 | 0.00 | 0 | 0.00 | 0 | 0.00 | 0 | 0.00 | 4 | 0.05 | 1 | 0.20 | 0 | 0.00 | 0 | 0.00 | 4 | 0.01 | 1 | 0.06 |  |
| Gordoniaceae | 0 | 0.00 | 0 | 0.00 | 0 | 0.00 | 0 | 0.00 | 0 | 0.00 | 0 | 0.00 | 4 | 0.05 | 1 | 0.20 | 0 | 0.00 | 0 | 0.00 | 4 | 0.01 | 1 | 0.06 |  |
| Methylocystaceae | 0 | 0.00 | 0 | 0.00 | 0 | 0.00 | 0 | 0.00 | 0 | 0.00 | 0 | 0.00 | 4 | 0.05 | 1 | 0.20 | 0 | 0.00 | 0 | 0.00 | 4 | 0.01 | 1 | 0.06 |  |
| Pelagibacteraceae | 1 | 0.03 | 1 | 0.22 | 0 | 0.00 | 0 | 0.00 | 3 | 0.03 | 2 | 0.36 | 0 | 0.00 | 0 | 0.00 | 0 | 0.00 | 0 | 0.00 | 4 | 0.01 | 2 | 0.13 |  |
| Rickettsiaceae | 0 | 0.00 | 0 | 0.00 | 0 | 0.00 | 0 | 0.00 | 2 | 0.02 | 2 | 0.36 | 0 | 0.00 | 0 | 0.00 | 2 | 0.01 | 2 | 0.40 | 4 | 0.01 | 3 | 0.19 |  |
| AKIW874 | 0 | 0.00 | 0 | 0.00 | 3 | 0.03 | 1 | 0.16 | 0 | 0.00 | 0 | 0.00 | 0 | 0.00 | 0 | 0.00 | 0 | 0.00 | 0 | 0.00 | 3 | 0.01 | 1 | 0.06 |  |
| Bacteriovoracaceae | 0 | 0.00 | 0 | 0.00 | 1 | 0.01 | 1 | 0.16 | 0 | 0.00 | 0 | 0.00 | 2 | 0.03 | 1 | 0.20 | 0 | 0.00 | 0 | 0.00 | 3 | 0.01 | 2 | 0.13 |  |
| Coprobacillaceae | 0 | 0.00 | 0 | 0.00 | 0 | 0.00 | 0 | 0.00 | 0 | 0.00 | 0 | 0.00 | 0 | 0.00 | 0 | 0.00 | 3 | 0.02 | 1 | 0.20 | 3 | 0.01 | 1 | 0.06 |  |
| Coriobacteriaceae | 0 | 0.00 | 0 | 0.00 | 0 | 0.00 | 0 | 0.00 | 0 | 0.00 | 0 | 0.00 | 0 | 0.00 | 0 | 0.00 | 3 | 0.02 | 1 | 0.20 | 3 | 0.01 | 1 | 0.06 |  |
| EB1017 | 0 | 0.00 | 0 | 0.00 | 0 | 0.00 | 0 | 0.00 | 3 | 0.03 | 0 | 0.00 | 0 | 0.00 | 0 | 0.00 | 0 | 0.00 | 0 | 0.00 | 3 | 0.01 | 1 | 0.06 |  |
| Bifidobacteriaceae | 0 | 0.00 | 0 | 0.00 | 2 | 0.02 | 1 | 0.16 | 0 | 0.00 | 0 | 0.00 | 0 | 0.00 | 1 | 0.20 | 0 | 0.00 | 0 | 0.00 | 2 | 0.00 | 1 | 0.06 |  |
| Dietziaceae | 0 | 0.00 | 0 | 0.00 | 0 | 0.00 | 0 | 0.00 | 0 | 0.00 | 0 | 0.00 | 2 | 0.03 | 1 | 0.20 | 0 | 0.00 | 0 | 0.00 | 2 | 0.00 | 1 | 0.06 |  |
| Enterococcaceae | 0 | 0.00 | 1 | 0.22 | 1 | 0.01 | 1 | 0.16 | 0 | 0.00 | 0 | 0.00 | 1 | 0.01 | 0 | 0.00 | 0 | 0.00 | 0 | 0.00 | 2 | 0.00 | 1 | 0.06 |  |
| Ignavibacteriaceae | 2 | 0.06 | 1 | 0.22 | 0 | 0.00 | 0 | 0.00 | 0 | 0.00 | 0 | 0.00 | 0 | 0.00 | 0 | 0.00 | 0 | 0.00 | 0 | 0.00 | 2 | 0.00 | 1 | 0.06 |  |
| Isosphaeraceae | 1 | 0.03 | 0 | 0.00 | 1 | 0.01 | 1 | 0.16 | 0 | 0.00 | 0 | 0.00 | 0 | 0.00 | 1 | 0.20 | 0 | 0.00 | 0 | 0.00 | 2 | 0.00 | 2 | 0.13 |  |
| Listeriaceae | 0 | 0.00 | 2 | 0.44 | 0 | 0.00 | 0 | 0.00 | 0 | 0.00 | 0 | 0.00 | 2 | 0.03 | 0 | 0.00 | 0 | 0.00 | 0 | 0.00 | 2 | 0.00 | 1 | 0.06 |  |
| Syntrophobacteraceae | 2 | 0.06 | 0 | 0.00 | 0 | 0.00 | 0 | 0.00 | 0 | 0.00 | 1 | 0.18 | 0 | 0.00 | 0 | 0.00 | 0 | 0.00 | 0 | 0.00 | 2 | 0.00 | 2 | 0.13 |  |
| Actinospicaceae | 0 | 0.00 | 0 | 0.00 | 0 | 0.00 | 0 | 0.00 | 1 | 0.01 | 0 | 0.00 | 0 | 0.00 | 1 | 0.20 | 0 | 0.00 | 0 | 0.00 | 1 | 0.00 | 1 | 0.06 |  |
| AEGEAN_112 | 0 | 0.00 | 0 | 0.00 | 0 | 0.00 | 0 | 0.00 | 0 | 0.00 | 0 | 0.00 | 1 | 0.01 | 0 | 0.00 | 0 | 0.00 | 0 | 0.00 | 1 | 0.00 | 1 | 0.06 |  |
| Amoebophilaceae | 0 | 0.00 | 0 | 0.00 | 1 | 0.01 | 1 | 0.16 | 0 | 0.00 | 0 | 0.00 | 0 | 0.00 | 1 | 0.20 | 0 | 0.00 | 0 | 0.00 | 1 | 0.00 | 1 | 0.06 |  |
| Christensenellaceae | 0 | 0.00 | 0 | 0.00 | 0 | 0.00 | 0 | 0.00 | 0 | 0.00 | 0 | 0.00 | 1 | 0.01 | 0 | 0.00 | 0 | 0.00 | 1 | 0.20 | 1 | 0.00 | 1 | 0.06 |  |
| Cryomorphaceae | 0 | 0.00 | 0 | 0.00 | 1 | 0.01 | 1 | 0.16 | 0 | 0.00 | 0 | 0.00 | 0 | 0.00 | 0 | 0.00 | 0 | 0.00 | 0 | 0.00 | 1 | 0.00 | 1 | 0.06 |  |
| Deinococcaceae | 0 | 0.00 | 0 | 0.00 | 0 | 0.00 | 0 | 0.00 | 0 | 0.00 | 0 | 0.00 | 0 | 0.00 | 0 | 0.00 | 1 | 0.01 | 1 | 0.20 | 1 | 0.00 | 1 | 0.06 |  |
| Geobacteraceae | 1 | 0.03 | 1 | 0.22 | 0 | 0.00 | 0 | 0.00 | 0 | 0.00 | 0 | 0.00 | 0 | 0.00 | 0 | 0.00 | 0 | 0.00 | 0 | 0.00 | 1 | 0.00 | 1 | 0.06 |  |
| Glycomycetaceae | 0 | 0.00 | 0 | 0.00 | 1 | 0.01 | 1 | 0.16 | 0 | 0.00 | 0 | 0.00 | 0 | 0.00 | 0 | 0.00 | 0 | 0.00 | 0 | 0.00 | 1 | 0.00 | 1 | 0.06 |  |
| Legionellaceae | 0 | 0.00 | 0 | 0.00 | 1 | 0.01 | 1 | 0.16 | 0 | 0.00 | 0 | 0.00 | 0 | 0.00 | 0 | 0.00 | 0 | 0.00 | 0 | 0.00 | 1 | 0.00 | 1 | 0.06 |  |
| Microthrixaceae | 1 | 0.03 | 1 | 0.22 | 0 | 0.00 | 0 | 0.00 | 0 | 0.00 | 0 | 0.00 | 0 | 0.00 | 0 | 0.00 | 0 | 0.00 | 0 | 0.00 | 1 | 0.00 | 1 | 0.06 |  |
| Nocardiopsaceae | 1 | 0.03 | 1 | 0.22 | 0 | 0.00 | 0 | 0.00 | 0 | 0.00 | 0 | 0.00 | 0 | 0.00 | 0 | 0.00 | 0 | 0.00 | 0 | 0.00 | 1 | 0.00 | 1 | 0.06 |  |
| Rubrobacteraceae | 0 | 0.00 | 0 | 0.00 | 1 | 0.01 | 1 | 0.16 | 0 | 0.00 | 0 | 0.00 | 0 | 0.00 | 0 | 0.00 | 0 | 0.00 | 0 | 0.00 | 1 | 0.00 | 1 | 0.06 |  |
| Shewanellaceae | 0 | 0.00 | 0 | 0.00 | 0 | 0.00 | 0 | 0.00 | 0 | 0.00 | 0 | 0.00 | 0 | 0.00 | 0 | 0.00 | 1 | 0.01 | 1 | 0.20 | 1 | 0.00 | 1 | 0.06 |  |
| Thermaceae | 0 | 0.00 | 0 | 0.00 | 0 | 0.00 | 0 | 0.00 | 0 | 0.00 | 0 | 0.00 | 1 | 0.01 | 1 | 0.20 | 0 | 0.00 | 0 | 0.00 | 1 | 0.00 | 1 | 0.06 |  |
| Thermomonosporaceae | 1 | 0.03 | 1 | 0.22 | 0 | 0.00 | 0 | 0.00 | 0 | 0.00 | 0 | 0.00 | 0 | 0.00 | 0 | 0.00 | 0 | 0.00 | 0 | 0.00 | 1 | 0.00 | 1 | 0.06 |  |
| Turicibacteraceae | 0 | 0.00 | 0 | 0.00 | 0 | 0.00 | 0 | 0.00 | 0 | 0.00 | 0 | 0.00 | 0 | 0.00 | 0 | 0.00 | 1 | 0.01 | 1 | 0.20 | 1 | 0.00 | 1 | 0.06 |  |
| Total | 3430 |  | 458 |  | 9368 |  | 624 |  | 10139 |  | 549 |  | 7582 |  | 491 |  | 16043 |  | 503 |  | 46562 |  | 1547 |  |  |

**Supplementary Table S3**. Community structure of leaf endophytic bacteria at genus level

| Genus | OUT | | | | | | | | | | | | Reads | | | | | | | | | | | |
| --- | --- | --- | --- | --- | --- | --- | --- | --- | --- | --- | --- | --- | --- | --- | --- | --- | --- | --- | --- | --- | --- | --- | --- | --- |
|  | C-0 | % | C-2 | % | C-4 | % | F-2 | % | F-4 | % | Total | % | C-0 |  | C-2 |  | C-4 |  | F-2 |  | F-4 |  | Total |  |
| A4 | 0 | 0.00 | 9 | 2.47 | 0 | 0.00 | 1 | 0.35 | 1 | 0.34 | 11 | 1.35 | 0 | 0.00 | 37 | 0.36 | 0 | 0.00 | 6 | 0.07 | 9 | 0.05 | 52 | 0.11 |
| *Achromobacter* | 1 | 0.44 | 1 | 0.27 | 0 | 0.00 | 0 | 0.00 | 1 | 0.34 | 1 | 0.12 | 1 | 0.03 | 3 | 0.03 | 0 | 0.00 | 0 | 0.00 | 2 | 0.01 | 6 | 0.01 |
| *Acidovorax* | 1 | 0.44 | 0 | 0.00 | 1 | 0.35 | 1 | 0.35 | 0 | 0.00 | 2 | 0.25 | 1 | 0.03 | 0 | 0.00 | 3 | 0.03 | 2 | 0.02 | 0 | 0.00 | 6 | 0.01 |
| *Acinetobacter* | 11 | 4.80 | 10 | 2.75 | 5 | 1.74 | 12 | 4.24 | 12 | 4.14 | 31 | 3.81 | 111 | 3.02 | 99 | 0.97 | 28 | 0.26 | 61 | 0.74 | 315 | 1.92 | 614 | 1.25 |
| *Actinomadura* | 1 | 0.44 | 0 | 0.00 | 0 | 0.00 | 0 | 0.00 | 0 | 0.00 | 1 | 0.12 | 1 | 0.03 | 0 | 0.00 | 0 | 0.00 | 0 | 0.00 | 0 | 0.00 | 1 | 0.00 |
| *Actinomycetospora* | 0 | 0.00 | 2 | 0.55 | 2 | 0.70 | 1 | 0.35 | 1 | 0.34 | 3 | 0.37 | 0 | 0.00 | 12 | 0.12 | 5 | 0.05 | 3 | 0.04 | 1 | 0.01 | 21 | 0.04 |
| *Adhaeribacter* | 0 | 0.00 | 1 | 0.27 | 0 | 0.00 | 0 | 0.00 | 0 | 0.00 | 1 | 0.12 | 0 | 0.00 | 2 | 0.02 | 0 | 0.00 | 0 | 0.00 | 0 | 0.00 | 2 | 0.00 |
| *Aerococcus* | 0 | 0.00 | 0 | 0.00 | 0 | 0.00 | 1 | 0.35 | 1 | 0.34 | 1 | 0.12 | 0 | 0.00 | 0 | 0.00 | 0 | 0.00 | 3 | 0.04 | 1 | 0.01 | 4 | 0.01 |
| *Aeromicrobium* | 1 | 0.44 | 1 | 0.27 | 1 | 0.35 | 0 | 0.00 | 1 | 0.34 | 2 | 0.25 | 5 | 0.14 | 7 | 0.07 | 2 | 0.02 | 0 | 0.00 | 4 | 0.02 | 18 | 0.04 |
| *Afifella* | 0 | 0.00 | 0 | 0.00 | 0 | 0.00 | 0 | 0.00 | 1 | 0.34 | 1 | 0.12 | 0 | 0.00 | 0 | 0.00 | 0 | 0.00 | 0 | 0.00 | 5 | 0.03 | 5 | 0.01 |
| *Agrobacterium* | 9 | 3.93 | 3 | 0.82 | 4 | 1.39 | 1 | 0.35 | 2 | 0.69 | 10 | 1.23 | 454 | 12.37 | 34 | 0.33 | 142 | 1.32 | 10 | 0.12 | 49 | 0.30 | 689 | 1.40 |
| *Agromyces* | 0 | 0.00 | 3 | 0.82 | 0 | 0.00 | 2 | 0.71 | 0 | 0.00 | 3 | 0.37 | 0 | 0.00 | 4 | 0.04 | 0 | 0.00 | 5 | 0.06 | 0 | 0.00 | 9 | 0.02 |
| *Allobaculum* | 0 | 0.00 | 2 | 0.55 | 0 | 0.00 | 0 | 0.00 | 0 | 0.00 | 2 | 0.25 | 0 | 0.00 | 6 | 0.06 | 0 | 0.00 | 0 | 0.00 | 0 | 0.00 | 6 | 0.01 |
| *Amycolatopsis* | 0 | 0.00 | 3 | 0.82 | 0 | 0.00 | 2 | 0.71 | 0 | 0.00 | 3 | 0.37 | 0 | 0.00 | 17 | 0.17 | 0 | 0.00 | 22 | 0.27 | 0 | 0.00 | 39 | 0.08 |
| *Anoxybacillus* | 0 | 0.00 | 1 | 0.27 | 1 | 0.35 | 1 | 0.35 | 0 | 0.00 | 1 | 0.12 | 0 | 0.00 | 4 | 0.04 | 2 | 0.02 | 11 | 0.13 | 0 | 0.00 | 17 | 0.03 |
| *Aquicella* | 0 | 0.00 | 1 | 0.27 | 1 | 0.35 | 0 | 0.00 | 0 | 0.00 | 2 | 0.25 | 0 | 0.00 | 2 | 0.02 | 3 | 0.03 | 0 | 0.00 | 0 | 0.00 | 5 | 0.01 |
| *Arsenicicoccus* | 0 | 0.00 | 0 | 0.00 | 0 | 0.00 | 0 | 0.00 | 1 | 0.34 | 1 | 0.12 | 0 | 0.00 | 0 | 0.00 | 0 | 0.00 | 0 | 0.00 | 4 | 0.02 | 4 | 0.01 |
| *Arthrobacter* | 3 | 1.31 | 0 | 0.00 | 1 | 0.35 | 1 | 0.35 | 1 | 0.34 | 3 | 0.37 | 16 | 0.44 | 0 | 0.00 | 2 | 0.02 | 1 | 0.01 | 1 | 0.01 | 20 | 0.04 |
| *Azohydromonas* | 1 | 0.44 | 0 | 0.00 | 0 | 0.00 | 0 | 0.00 | 0 | 0.00 | 1 | 0.12 | 1 | 0.03 | 0 | 0.00 | 0 | 0.00 | 0 | 0.00 | 0 | 0.00 | 1 | 0.00 |
| *Azospirillum* | 0 | 0.00 | 1 | 0.27 | 0 | 0.00 | 0 | 0.00 | 0 | 0.00 | 1 | 0.12 | 0 | 0.00 | 2 | 0.02 | 0 | 0.00 | 0 | 0.00 | 0 | 0.00 | 2 | 0.00 |
| *Bacillus* | 4 | 1.75 | 5 | 1.37 | 1 | 0.35 | 5 | 1.77 | 1 | 0.34 | 7 | 0.86 | 53 | 1.44 | 34 | 0.33 | 1 | 0.01 | 32 | 0.39 | 3 | 0.02 | 123 | 0.25 |
| *Bacteroides* | 0 | 0.00 | 2 | 0.55 | 0 | 0.00 | 1 | 0.35 | 0 | 0.00 | 3 | 0.37 | 0 | 0.00 | 10 | 0.10 | 0 | 0.00 | 2 | 0.02 | 0 | 0.00 | 12 | 0.02 |
| *Balneimonas* | 2 | 0.87 | 1 | 0.27 | 0 | 0.00 | 1 | 0.35 | 0 | 0.00 | 2 | 0.25 | 2 | 0.05 | 17 | 0.17 | 0 | 0.00 | 7 | 0.09 | 0 | 0.00 | 26 | 0.05 |
| *Bdellovibrio* | 0 | 0.00 | 1 | 0.27 | 2 | 0.70 | 3 | 1.06 | 1 | 0.34 | 4 | 0.49 | 0 | 0.00 | 53 | 0.52 | 9 | 0.08 | 18 | 0.22 | 6 | 0.04 | 86 | 0.17 |
| *Bifidobacterium* | 0 | 0.00 | 1 | 0.27 | 0 | 0.00 | 0 | 0.00 | 0 | 0.00 | 1 | 0.12 | 0 | 0.00 | 2 | 0.02 | 0 | 0.00 | 0 | 0.00 | 0 | 0.00 | 2 | 0.00 |
| *Blautia* | 2 | 0.87 | 2 | 0.55 | 0 | 0.00 | 0 | 0.00 | 1 | 0.34 | 3 | 0.37 | 4 | 0.11 | 50 | 0.49 | 0 | 0.00 | 0 | 0.00 | 14 | 0.09 | 68 | 0.14 |
| *Bosea* | 1 | 0.44 | 0 | 0.00 | 0 | 0.00 | 0 | 0.00 | 0 | 0.00 | 1 | 0.12 | 1 | 0.03 | 0 | 0.00 | 0 | 0.00 | 0 | 0.00 | 0 | 0.00 | 1 | 0.00 |
| *Brachybacterium* | 1 | 0.44 | 1 | 0.27 | 1 | 0.35 | 3 | 1.06 | 1 | 0.34 | 4 | 0.49 | 5 | 0.14 | 13 | 0.13 | 1 | 0.01 | 23 | 0.28 | 4 | 0.02 | 46 | 0.09 |
| *Brevibacterium* | 0 | 0.00 | 1 | 0.27 | 0 | 0.00 | 0 | 0.00 | 1 | 0.34 | 2 | 0.25 | 0 | 0.00 | 2 | 0.02 | 0 | 0.00 | 0 | 0.00 | 2 | 0.01 | 4 | 0.01 |
| *Brevundimonas* | 1 | 0.44 | 2 | 0.55 | 5 | 1.74 | 2 | 0.71 | 2 | 0.69 | 7 | 0.86 | 1 | 0.03 | 7 | 0.07 | 28 | 0.26 | 42 | 0.51 | 8 | 0.05 | 86 | 0.17 |
| *Brochothrix* | 0 | 0.00 | 0 | 0.00 | 0 | 0.00 | 1 | 0.35 | 0 | 0.00 | 1 | 0.12 | 0 | 0.00 | 0 | 0.00 | 0 | 0.00 | 2 | 0.02 | 0 | 0.00 | 2 | 0.00 |
| *Buchnera* | 0 | 0.00 | 1 | 0.27 | 0 | 0.00 | 0 | 0.00 | 1 | 0.34 | 1 | 0.12 | 0 | 0.00 | 12 | 0.12 | 0 | 0.00 | 0 | 0.00 | 1 | 0.01 | 13 | 0.03 |
| *Burkholderia* | 3 | 1.31 | 0 | 0.00 | 3 | 1.05 | 1 | 0.35 | 1 | 0.34 | 7 | 0.86 | 5 | 0.14 | 0 | 0.00 | 3 | 0.03 | 4 | 0.05 | 1 | 0.01 | 13 | 0.03 |
| *Campylobacter* | 0 | 0.00 | 1 | 0.27 | 0 | 0.00 | 0 | 0.00 | 0 | 0.00 | 1 | 0.12 | 0 | 0.00 | 7 | 0.07 | 0 | 0.00 | 0 | 0.00 | 0 | 0.00 | 7 | 0.01 |
| *Candidatus Koribacter* | 1 | 0.44 | 1 | 0.27 | 0 | 0.00 | 0 | 0.00 | 0 | 0.00 | 2 | 0.25 | 1 | 0.03 | 13 | 0.13 | 0 | 0.00 | 0 | 0.00 | 0 | 0.00 | 14 | 0.03 |
| *Candidatus Phlomobacter* | 0 | 0.00 | 1 | 0.27 | 0 | 0.00 | 0 | 0.00 | 0 | 0.00 | 1 | 0.12 | 0 | 0.00 | 2 | 0.02 | 0 | 0.00 | 0 | 0.00 | 0 | 0.00 | 2 | 0.00 |
| *Candidatus Portiera* | 0 | 0.00 | 0 | 0.00 | 0 | 0.00 | 0 | 0.00 | 2 | 0.69 | 2 | 0.25 | 0 | 0.00 | 0 | 0.00 | 0 | 0.00 | 0 | 0.00 | 3 | 0.02 | 3 | 0.01 |
| *Candidatus Solibacter* | 4 | 1.75 | 7 | 1.92 | 3 | 1.05 | 2 | 0.71 | 1 | 0.34 | 13 | 1.60 | 6 | 0.16 | 55 | 0.54 | 3 | 0.03 | 22 | 0.27 | 8 | 0.05 | 94 | 0.19 |
| *Catellatospora* | 0 | 0.00 | 1 | 0.27 | 0 | 0.00 | 0 | 0.00 | 0 | 0.00 | 1 | 0.12 | 0 | 0.00 | 2 | 0.02 | 0 | 0.00 | 0 | 0.00 | 0 | 0.00 | 2 | 0.00 |
| *Catenulispora* | 0 | 0.00 | 1 | 0.27 | 0 | 0.00 | 0 | 0.00 | 0 | 0.00 | 1 | 0.12 | 0 | 0.00 | 10 | 0.10 | 0 | 0.00 | 0 | 0.00 | 0 | 0.00 | 10 | 0.02 |
| *Caulobacter* | 1 | 0.44 | 2 | 0.55 | 1 | 0.35 | 1 | 0.35 | 1 | 0.34 | 2 | 0.25 | 7 | 0.19 | 26 | 0.25 | 1 | 0.01 | 14 | 0.17 | 16 | 0.10 | 64 | 0.13 |
| *Cellulomonas* | 1 | 0.44 | 1 | 0.27 | 1 | 0.35 | 1 | 0.35 | 1 | 0.34 | 1 | 0.12 | 6 | 0.16 | 19 | 0.19 | 4 | 0.04 | 9 | 0.11 | 1 | 0.01 | 39 | 0.08 |
| *Cellvibrio* | 0 | 0.00 | 1 | 0.27 | 0 | 0.00 | 0 | 0.00 | 1 | 0.34 | 2 | 0.25 | 0 | 0.00 | 6 | 0.06 | 0 | 0.00 | 0 | 0.00 | 3 | 0.02 | 9 | 0.02 |
| *Chitinophaga* | 0 | 0.00 | 1 | 0.27 | 0 | 0.00 | 0 | 0.00 | 0 | 0.00 | 1 | 0.12 | 0 | 0.00 | 5 | 0.05 | 0 | 0.00 | 0 | 0.00 | 0 | 0.00 | 5 | 0.01 |
| *Chryseobacterium* | 2 | 0.87 | 3 | 0.82 | 1 | 0.35 | 0 | 0.00 | 3 | 1.03 | 6 | 0.74 | 33 | 0.90 | 11 | 0.11 | 5 | 0.05 | 0 | 0.00 | 38 | 0.23 | 87 | 0.18 |
| *Citrobacter* | 0 | 0.00 | 1 | 0.27 | 1 | 0.35 | 2 | 0.71 | 1 | 0.34 | 2 | 0.25 | 0 | 0.00 | 2 | 0.02 | 1 | 0.01 | 8 | 0.10 | 3 | 0.02 | 14 | 0.03 |
| *Clostridium* | 2 | 0.87 | 1 | 0.27 | 0 | 0.00 | 0 | 0.00 | 1 | 0.34 | 3 | 0.37 | 3 | 0.08 | 3 | 0.03 | 0 | 0.00 | 0 | 0.00 | 1 | 0.01 | 7 | 0.01 |
| *Comamonas* | 2 | 0.87 | 0 | 0.00 | 1 | 0.35 | 1 | 0.35 | 1 | 0.34 | 2 | 0.25 | 5 | 0.14 | 0 | 0.00 | 1 | 0.01 | 7 | 0.09 | 5 | 0.03 | 18 | 0.04 |
| *Corynebacterium* | 0 | 0.00 | 3 | 0.82 | 2 | 0.70 | 4 | 1.41 | 3 | 1.03 | 7 | 0.86 | 0 | 0.00 | 6 | 0.06 | 5 | 0.05 | 27 | 0.33 | 6 | 0.04 | 44 | 0.09 |
| *Cryocola* | 1 | 0.44 | 0 | 0.00 | 0 | 0.00 | 0 | 0.00 | 0 | 0.00 | 1 | 0.12 | 1 | 0.03 | 0 | 0.00 | 0 | 0.00 | 0 | 0.00 | 0 | 0.00 | 1 | 0.00 |
| *Deinococcus* | 0 | 0.00 | 0 | 0.00 | 0 | 0.00 | 0 | 0.00 | 1 | 0.34 | 1 | 0.12 | 0 | 0.00 | 0 | 0.00 | 0 | 0.00 | 0 | 0.00 | 1 | 0.01 | 1 | 0.00 |
| *Delftia* | 1 | 0.44 | 1 | 0.27 | 1 | 0.35 | 2 | 0.71 | 1 | 0.34 | 2 | 0.25 | 5 | 0.14 | 2 | 0.02 | 1 | 0.01 | 43 | 0.52 | 8 | 0.05 | 59 | 0.12 |
| *Desemzia* | 0 | 0.00 | 0 | 0.00 | 0 | 0.00 | 1 | 0.35 | 0 | 0.00 | 1 | 0.12 | 0 | 0.00 | 0 | 0.00 | 0 | 0.00 | 8 | 0.10 | 0 | 0.00 | 8 | 0.02 |
| *Desulfovibrio* | 0 | 0.00 | 3 | 0.82 | 1 | 0.35 | 1 | 0.35 | 2 | 0.69 | 3 | 0.37 | 0 | 0.00 | 15 | 0.15 | 1 | 0.01 | 24 | 0.29 | 23 | 0.14 | 63 | 0.13 |
| *Devosia* | 6 | 2.62 | 3 | 0.82 | 2 | 0.70 | 5 | 1.77 | 1 | 0.34 | 7 | 0.86 | 15 | 0.41 | 15 | 0.15 | 7 | 0.07 | 15 | 0.18 | 2 | 0.01 | 54 | 0.11 |
| *Dietzia* | 0 | 0.00 | 0 | 0.00 | 0 | 0.00 | 1 | 0.35 | 0 | 0.00 | 1 | 0.12 | 0 | 0.00 | 0 | 0.00 | 0 | 0.00 | 2 | 0.02 | 0 | 0.00 | 2 | 0.00 |
| *Dyadobacter* | 3 | 1.31 | 3 | 0.82 | 0 | 0.00 | 1 | 0.35 | 1 | 0.34 | 4 | 0.49 | 5 | 0.14 | 26 | 0.25 | 0 | 0.00 | 2 | 0.02 | 6 | 0.04 | 39 | 0.08 |
| *Enhydrobacter* | 1 | 0.44 | 1 | 0.27 | 1 | 0.35 | 1 | 0.35 | 2 | 0.69 | 2 | 0.25 | 1 | 0.03 | 1 | 0.01 | 1 | 0.01 | 8 | 0.10 | 9 | 0.05 | 20 | 0.04 |
| *Enterobacter* | 2 | 0.87 | 2 | 0.55 | 1 | 0.35 | 0 | 0.00 | 2 | 0.69 | 4 | 0.49 | 3 | 0.08 | 2 | 0.02 | 2 | 0.02 | 0 | 0.00 | 6 | 0.04 | 13 | 0.03 |
| *Erwinia* | 8 | 3.49 | 5 | 1.37 | 15 | 5.23 | 2 | 0.71 | 5 | 1.72 | 19 | 2.34 | 267 | 7.28 | 64 | 0.63 | 215 | 2.00 | 15 | 0.18 | 31 | 0.19 | 592 | 1.20 |
| *Erythromicrobium* | 0 | 0.00 | 0 | 0.00 | 0 | 0.00 | 1 | 0.35 | 0 | 0.00 | 1 | 0.12 | 0 | 0.00 | 0 | 0.00 | 0 | 0.00 | 1 | 0.01 | 0 | 0.00 | 1 | 0.00 |
| *Escherichia* | 1 | 0.44 | 3 | 0.82 | 3 | 1.05 | 4 | 1.41 | 4 | 1.38 | 5 | 0.62 | 2 | 0.05 | 108 | 1.06 | 20 | 0.19 | 598 | 7.28 | 127 | 0.77 | 855 | 1.73 |
| *Eubacterium* | 0 | 0.00 | 1 | 0.27 | 0 | 0.00 | 0 | 0.00 | 0 | 0.00 | 1 | 0.12 | 0 | 0.00 | 10 | 0.10 | 0 | 0.00 | 0 | 0.00 | 0 | 0.00 | 10 | 0.02 |
| *Exiguobacterium* | 0 | 0.00 | 7 | 1.92 | 0 | 0.00 | 4 | 1.41 | 0 | 0.00 | 7 | 0.86 | 0 | 0.00 | 163 | 1.59 | 0 | 0.00 | 13 | 0.16 | 0 | 0.00 | 176 | 0.36 |
| *Fimbriimonas* | 0 | 0.00 | 0 | 0.00 | 0 | 0.00 | 1 | 0.35 | 0 | 0.00 | 1 | 0.12 | 0 | 0.00 | 0 | 0.00 | 0 | 0.00 | 4 | 0.05 | 0 | 0.00 | 4 | 0.01 |
| *Flavisolibacter* | 0 | 0.00 | 5 | 1.37 | 0 | 0.00 | 0 | 0.00 | 0 | 0.00 | 5 | 0.62 | 0 | 0.00 | 20 | 0.20 | 0 | 0.00 | 0 | 0.00 | 0 | 0.00 | 20 | 0.04 |
| *Flavobacterium* | 6 | 2.62 | 5 | 1.37 | 0 | 0.00 | 2 | 0.71 | 2 | 0.69 | 13 | 1.60 | 11 | 0.30 | 13 | 0.13 | 0 | 0.00 | 3 | 0.04 | 5 | 0.03 | 32 | 0.06 |
| *Geobacillus* | 0 | 0.00 | 0 | 0.00 | 0 | 0.00 | 1 | 0.35 | 0 | 0.00 | 1 | 0.12 | 0 | 0.00 | 0 | 0.00 | 0 | 0.00 | 1 | 0.01 | 0 | 0.00 | 1 | 0.00 |
| *Geobacter* | 1 | 0.44 | 0 | 0.00 | 0 | 0.00 | 0 | 0.00 | 0 | 0.00 | 1 | 0.12 | 1 | 0.03 | 0 | 0.00 | 0 | 0.00 | 0 | 0.00 | 0 | 0.00 | 1 | 0.00 |
| *Geodermatophilus* | 2 | 0.87 | 4 | 1.10 | 3 | 1.05 | 2 | 0.71 | 3 | 1.03 | 7 | 0.86 | 6 | 0.16 | 121 | 1.18 | 38 | 0.35 | 45 | 0.55 | 184 | 1.12 | 394 | 0.80 |
| *Glycomyces* | 0 | 0.00 | 1 | 0.27 | 0 | 0.00 | 0 | 0.00 | 0 | 0.00 | 1 | 0.12 | 0 | 0.00 | 1 | 0.01 | 0 | 0.00 | 0 | 0.00 | 0 | 0.00 | 1 | 0.00 |
| *Gordonia* | 0 | 0.00 | 0 | 0.00 | 0 | 0.00 | 1 | 0.35 | 0 | 0.00 | 1 | 0.12 | 0 | 0.00 | 0 | 0.00 | 0 | 0.00 | 4 | 0.05 | 0 | 0.00 | 4 | 0.01 |
| *Halomonas* | 0 | 0.00 | 1 | 0.27 | 0 | 0.00 | 0 | 0.00 | 1 | 0.34 | 2 | 0.25 | 0 | 0.00 | 4 | 0.04 | 0 | 0.00 | 0 | 0.00 | 4 | 0.02 | 8 | 0.02 |
| *Herbaspirillum* | 0 | 0.00 | 0 | 0.00 | 0 | 0.00 | 2 | 0.71 | 0 | 0.00 | 2 | 0.25 | 0 | 0.00 | 0 | 0.00 | 0 | 0.00 | 6 | 0.07 | 0 | 0.00 | 6 | 0.01 |
| *Hydrogenophaga* | 1 | 0.44 | 2 | 0.55 | 0 | 0.00 | 1 | 0.35 | 2 | 0.69 | 3 | 0.37 | 1 | 0.03 | 28 | 0.27 | 0 | 0.00 | 3 | 0.04 | 2 | 0.01 | 34 | 0.07 |
| *Hymenobacter* | 0 | 0.00 | 17 | 4.67 | 7 | 2.44 | 11 | 3.89 | 7 | 2.41 | 25 | 3.08 | 0 | 0.00 | 190 | 1.86 | 30 | 0.28 | 68 | 0.83 | 248 | 1.51 | 536 | 1.09 |
| *Hyphomicrobium* | 1 | 0.44 | 0 | 0.00 | 0 | 0.00 | 0 | 0.00 | 0 | 0.00 | 1 | 0.12 | 1 | 0.03 | 0 | 0.00 | 0 | 0.00 | 0 | 0.00 | 0 | 0.00 | 1 | 0.00 |
| *Hyphomonas* | 0 | 0.00 | 0 | 0.00 | 1 | 0.35 | 1 | 0.35 | 0 | 0.00 | 1 | 0.12 | 0 | 0.00 | 0 | 0.00 | 1 | 0.01 | 1 | 0.01 | 0 | 0.00 | 2 | 0.00 |
| *Iamia* | 2 | 0.87 | 1 | 0.27 | 0 | 0.00 | 0 | 0.00 | 1 | 0.34 | 2 | 0.25 | 7 | 0.19 | 4 | 0.04 | 0 | 0.00 | 0 | 0.00 | 11 | 0.07 | 22 | 0.04 |
| *Janthinobacterium* | 0 | 0.00 | 2 | 0.55 | 3 | 1.05 | 1 | 0.35 | 1 | 0.34 | 3 | 0.37 | 0 | 0.00 | 43 | 0.42 | 19 | 0.18 | 9 | 0.11 | 5 | 0.03 | 76 | 0.15 |
| *Jeotgalicoccus* | 0 | 0.00 | 0 | 0.00 | 0 | 0.00 | 0 | 0.00 | 1 | 0.34 | 1 | 0.12 | 0 | 0.00 | 0 | 0.00 | 0 | 0.00 | 0 | 0.00 | 1 | 0.01 | 1 | 0.00 |
| *Kaistia* | 1 | 0.44 | 1 | 0.27 | 0 | 0.00 | 0 | 0.00 | 0 | 0.00 | 1 | 0.12 | 4 | 0.11 | 1 | 0.01 | 0 | 0.00 | 0 | 0.00 | 0 | 0.00 | 5 | 0.01 |
| *Kaistobacter* | 4 | 1.75 | 5 | 1.37 | 3 | 1.05 | 3 | 1.06 | 3 | 1.03 | 8 | 0.98 | 11 | 0.30 | 77 | 0.75 | 12 | 0.11 | 28 | 0.34 | 8 | 0.05 | 136 | 0.28 |
| *Kineococcus* | 1 | 0.44 | 4 | 1.10 | 4 | 1.39 | 2 | 0.71 | 4 | 1.38 | 7 | 0.86 | 3 | 0.08 | 187 | 1.83 | 23 | 0.21 | 112 | 1.36 | 71 | 0.43 | 396 | 0.80 |
| *Kribbella* | 0 | 0.00 | 1 | 0.27 | 0 | 0.00 | 1 | 0.35 | 0 | 0.00 | 1 | 0.12 | 0 | 0.00 | 16 | 0.16 | 0 | 0.00 | 2 | 0.02 | 0 | 0.00 | 18 | 0.04 |
| *Lactobacillus* | 0 | 0.00 | 2 | 0.55 | 2 | 0.70 | 0 | 0.00 | 0 | 0.00 | 2 | 0.25 | 0 | 0.00 | 2 | 0.02 | 2 | 0.02 | 0 | 0.00 | 0 | 0.00 | 4 | 0.01 |
| *Lactococcus* | 0 | 0.00 | 2 | 0.55 | 1 | 0.35 | 2 | 0.71 | 2 | 0.69 | 3 | 0.37 | 0 | 0.00 | 13 | 0.13 | 1 | 0.01 | 14 | 0.17 | 7 | 0.04 | 35 | 0.07 |
| *Legionella* | 0 | 0.00 | 1 | 0.27 | 0 | 0.00 | 0 | 0.00 | 0 | 0.00 | 1 | 0.12 | 0 | 0.00 | 1 | 0.01 | 0 | 0.00 | 0 | 0.00 | 0 | 0.00 | 1 | 0.00 |
| *Limnobacter* | 0 | 0.00 | 1 | 0.27 | 0 | 0.00 | 1 | 0.35 | 0 | 0.00 | 1 | 0.12 | 0 | 0.00 | 17 | 0.17 | 0 | 0.00 | 2 | 0.02 | 0 | 0.00 | 19 | 0.04 |
| *Limnohabitans* | 0 | 0.00 | 1 | 0.27 | 0 | 0.00 | 0 | 0.00 | 0 | 0.00 | 1 | 0.12 | 0 | 0.00 | 2 | 0.02 | 0 | 0.00 | 0 | 0.00 | 0 | 0.00 | 2 | 0.00 |
| *Luteibacter* | 3 | 1.31 | 0 | 0.00 | 0 | 0.00 | 1 | 0.35 | 1 | 0.34 | 3 | 0.37 | 3 | 0.08 | 0 | 0.00 | 0 | 0.00 | 1 | 0.01 | 2 | 0.01 | 6 | 0.01 |
| *Luteimonas* | 1 | 0.44 | 0 | 0.00 | 0 | 0.00 | 1 | 0.35 | 0 | 0.00 | 1 | 0.12 | 3 | 0.08 | 0 | 0.00 | 0 | 0.00 | 1 | 0.01 | 0 | 0.00 | 4 | 0.01 |
| *Lysobacter* | 1 | 0.44 | 2 | 0.55 | 1 | 0.35 | 1 | 0.35 | 1 | 0.34 | 4 | 0.49 | 2 | 0.05 | 7 | 0.07 | 1 | 0.01 | 4 | 0.05 | 4 | 0.02 | 18 | 0.04 |
| *Maricaulis* | 1 | 0.44 | 0 | 0.00 | 0 | 0.00 | 0 | 0.00 | 0 | 0.00 | 1 | 0.12 | 1 | 0.03 | 0 | 0.00 | 0 | 0.00 | 0 | 0.00 | 0 | 0.00 | 1 | 0.00 |
| *Marinomonas* | 0 | 0.00 | 1 | 0.27 | 2 | 0.70 | 1 | 0.35 | 1 | 0.34 | 2 | 0.25 | 0 | 0.00 | 4 | 0.04 | 3 | 0.03 | 2 | 0.02 | 2 | 0.01 | 11 | 0.02 |
| *Methylibium* | 0 | 0.00 | 2 | 0.55 | 0 | 0.00 | 1 | 0.35 | 2 | 0.69 | 3 | 0.37 | 0 | 0.00 | 4 | 0.04 | 0 | 0.00 | 1 | 0.01 | 3 | 0.02 | 8 | 0.02 |
| ***Methylobacterium*** | **14** | **6.11** | **32** | **8.79** | **39** | **13.59** | **37** | **13.07** | **25** | **8.62** | **68** | **8.36** | **174** | **4.74** | **1686** | **16.48** | **1063** | **9.89** | **1669** | **20.33** | **1137** | **6.92** | **5729** | **11.62** |
| *Methylopila* | 0 | 0.00 | 0 | 0.00 | 0 | 0.00 | 1 | 0.35 | 0 | 0.00 | 1 | 0.12 | 0 | 0.00 | 0 | 0.00 | 0 | 0.00 | 4 | 0.05 | 0 | 0.00 | 4 | 0.01 |
| *Methylotenera* | 0 | 0.00 | 2 | 0.55 | 0 | 0.00 | 2 | 0.71 | 0 | 0.00 | 4 | 0.49 | 0 | 0.00 | 10 | 0.10 | 0 | 0.00 | 6 | 0.07 | 0 | 0.00 | 16 | 0.03 |
| *Methyloversatilis* | 0 | 0.00 | 0 | 0.00 | 0 | 0.00 | 1 | 0.35 | 0 | 0.00 | 1 | 0.12 | 0 | 0.00 | 0 | 0.00 | 0 | 0.00 | 1 | 0.01 | 0 | 0.00 | 1 | 0.00 |
| *Microbacterium* | 1 | 0.44 | 0 | 0.00 | 0 | 0.00 | 1 | 0.35 | 1 | 0.34 | 1 | 0.12 | 1 | 0.03 | 0 | 0.00 | 0 | 0.00 | 1 | 0.01 | 10 | 0.06 | 12 | 0.02 |
| *Microbispora* | 1 | 0.44 | 1 | 0.27 | 1 | 0.35 | 2 | 0.71 | 1 | 0.34 | 2 | 0.25 | 13 | 0.35 | 3 | 0.03 | 1 | 0.01 | 3 | 0.04 | 4 | 0.02 | 24 | 0.05 |
| *Micrococcus* | 0 | 0.00 | 0 | 0.00 | 0 | 0.00 | 1 | 0.35 | 1 | 0.34 | 1 | 0.12 | 0 | 0.00 | 0 | 0.00 | 0 | 0.00 | 4 | 0.05 | 1 | 0.01 | 5 | 0.01 |
| *Modestobacter* | 1 | 0.44 | 1 | 0.27 | 0 | 0.00 | 1 | 0.35 | 0 | 0.00 | 1 | 0.12 | 1 | 0.03 | 4 | 0.04 | 0 | 0.00 | 2 | 0.02 | 0 | 0.00 | 7 | 0.01 |
| *Mycobacterium* | 2 | 0.87 | 3 | 0.82 | 2 | 0.70 | 2 | 0.71 | 2 | 0.69 | 6 | 0.74 | 2 | 0.05 | 8 | 0.08 | 2 | 0.02 | 3 | 0.04 | 5 | 0.03 | 20 | 0.04 |
| *Mycoplana* | 1 | 0.44 | 2 | 0.55 | 1 | 0.35 | 1 | 0.35 | 1 | 0.34 | 2 | 0.25 | 4 | 0.11 | 13 | 0.13 | 2 | 0.02 | 3 | 0.04 | 2 | 0.01 | 24 | 0.05 |
| *Myxococcus* | 0 | 0.00 | 1 | 0.27 | 0 | 0.00 | 0 | 0.00 | 0 | 0.00 | 1 | 0.12 | 0 | 0.00 | 3 | 0.03 | 0 | 0.00 | 0 | 0.00 | 0 | 0.00 | 3 | 0.01 |
| *Nesterenkonia* | 0 | 0.00 | 1 | 0.27 | 0 | 0.00 | 0 | 0.00 | 0 | 0.00 | 1 | 0.12 | 0 | 0.00 | 4 | 0.04 | 0 | 0.00 | 0 | 0.00 | 0 | 0.00 | 4 | 0.01 |
| *Nocardioides* | 5 | 2.18 | 9 | 2.47 | 2 | 0.70 | 4 | 1.41 | 5 | 1.72 | 13 | 1.60 | 20 | 0.54 | 271 | 2.65 | 8 | 0.07 | 101 | 1.23 | 48 | 0.29 | 448 | 0.91 |
| *Novosphingobium* | 7 | 3.06 | 2 | 0.55 | 3 | 1.05 | 1 | 0.35 | 4 | 1.38 | 12 | 1.48 | 131 | 3.57 | 8 | 0.08 | 44 | 0.41 | 2 | 0.02 | 157 | 0.96 | 342 | 0.69 |
| *Ochrobactrum* | 1 | 0.44 | 0 | 0.00 | 1 | 0.35 | 2 | 0.71 | 1 | 0.34 | 2 | 0.25 | 9 | 0.25 | 0 | 0.00 | 7 | 0.07 | 10 | 0.12 | 6 | 0.04 | 32 | 0.06 |
| *Oleibacter* | 0 | 0.00 | 0 | 0.00 | 0 | 0.00 | 0 | 0.00 | 1 | 0.34 | 1 | 0.12 | 0 | 0.00 | 0 | 0.00 | 0 | 0.00 | 0 | 0.00 | 3 | 0.02 | 3 | 0.01 |
| *Oscillospira* | 2 | 0.87 | 1 | 0.27 | 1 | 0.35 | 1 | 0.35 | 2 | 0.69 | 6 | 0.74 | 5 | 0.14 | 4 | 0.04 | 6 | 0.06 | 6 | 0.07 | 9 | 0.05 | 30 | 0.06 |
| *Paenibacillus* | 6 | 2.62 | 1 | 0.27 | 2 | 0.70 | 0 | 0.00 | 1 | 0.34 | 9 | 1.11 | 29 | 0.79 | 1 | 0.01 | 6 | 0.06 | 0 | 0.00 | 1 | 0.01 | 37 | 0.08 |
| *Parabacteroides* | 0 | 0.00 | 0 | 0.00 | 0 | 0.00 | 2 | 0.71 | 0 | 0.00 | 2 | 0.25 | 0 | 0.00 | 0 | 0.00 | 0 | 0.00 | 11 | 0.13 | 0 | 0.00 | 11 | 0.02 |
| *Paracoccus* | 2 | 0.87 | 4 | 1.10 | 1 | 0.35 | 2 | 0.71 | 1 | 0.34 | 4 | 0.49 | 9 | 0.25 | 37 | 0.36 | 1 | 0.01 | 12 | 0.15 | 1 | 0.01 | 60 | 0.12 |
| *Parapedobacter* | 1 | 0.44 | 0 | 0.00 | 0 | 0.00 | 1 | 0.35 | 1 | 0.34 | 2 | 0.25 | 2 | 0.05 | 0 | 0.00 | 0 | 0.00 | 13 | 0.16 | 3 | 0.02 | 18 | 0.04 |
| *Paraprevotella* | 0 | 0.00 | 0 | 0.00 | 0 | 0.00 | 0 | 0.00 | 1 | 0.34 | 1 | 0.12 | 0 | 0.00 | 0 | 0.00 | 0 | 0.00 | 0 | 0.00 | 4 | 0.02 | 4 | 0.01 |
| *Patulibacter* | 0 | 0.00 | 1 | 0.27 | 1 | 0.35 | 0 | 0.00 | 0 | 0.00 | 2 | 0.25 | 0 | 0.00 | 7 | 0.07 | 1 | 0.01 | 0 | 0.00 | 0 | 0.00 | 8 | 0.02 |
| *Pedobacter* | 2 | 0.87 | 1 | 0.27 | 0 | 0.00 | 0 | 0.00 | 0 | 0.00 | 3 | 0.37 | 4 | 0.11 | 8 | 0.08 | 0 | 0.00 | 0 | 0.00 | 0 | 0.00 | 12 | 0.02 |
| *Pelomonas* | 0 | 0.00 | 2 | 0.55 | 1 | 0.35 | 1 | 0.35 | 2 | 0.69 | 2 | 0.25 | 0 | 0.00 | 6 | 0.06 | 2 | 0.02 | 8 | 0.10 | 7 | 0.04 | 23 | 0.05 |
| *Peptoniphilus* | 0 | 0.00 | 0 | 0.00 | 0 | 0.00 | 1 | 0.35 | 0 | 0.00 | 1 | 0.12 | 0 | 0.00 | 0 | 0.00 | 0 | 0.00 | 1 | 0.01 | 0 | 0.00 | 1 | 0.00 |
| *Phaeospirillum* | 0 | 0.00 | 2 | 0.55 | 0 | 0.00 | 1 | 0.35 | 0 | 0.00 | 2 | 0.25 | 0 | 0.00 | 6 | 0.06 | 0 | 0.00 | 2 | 0.02 | 0 | 0.00 | 8 | 0.02 |
| *Phenylobacterium* | 1 | 0.44 | 3 | 0.82 | 0 | 0.00 | 1 | 0.35 | 0 | 0.00 | 3 | 0.37 | 6 | 0.16 | 8 | 0.08 | 0 | 0.00 | 6 | 0.07 | 0 | 0.00 | 20 | 0.04 |
| *Pilimelia* | 1 | 0.44 | 0 | 0.00 | 0 | 0.00 | 0 | 0.00 | 0 | 0.00 | 1 | 0.12 | 1 | 0.03 | 0 | 0.00 | 0 | 0.00 | 0 | 0.00 | 0 | 0.00 | 1 | 0.00 |
| *Pimelobacter* | 1 | 0.44 | 0 | 0.00 | 0 | 0.00 | 0 | 0.00 | 0 | 0.00 | 1 | 0.12 | 2 | 0.05 | 0 | 0.00 | 0 | 0.00 | 0 | 0.00 | 0 | 0.00 | 2 | 0.00 |
| *Planifilum* | 0 | 0.00 | 1 | 0.27 | 0 | 0.00 | 0 | 0.00 | 2 | 0.69 | 2 | 0.25 | 0 | 0.00 | 3 | 0.03 | 0 | 0.00 | 0 | 0.00 | 6 | 0.04 | 9 | 0.02 |
| *Planomicrobium* | 0 | 0.00 | 1 | 0.27 | 0 | 0.00 | 1 | 0.35 | 1 | 0.34 | 1 | 0.12 | 0 | 0.00 | 4 | 0.04 | 0 | 0.00 | 7 | 0.09 | 3 | 0.02 | 14 | 0.03 |
| *Pontibacter* | 1 | 0.44 | 1 | 0.27 | 0 | 0.00 | 1 | 0.35 | 0 | 0.00 | 3 | 0.37 | 1 | 0.03 | 2 | 0.02 | 0 | 0.00 | 1 | 0.01 | 0 | 0.00 | 4 | 0.01 |
| *Prevotella* | 0 | 0.00 | 0 | 0.00 | 0 | 0.00 | 1 | 0.35 | 1 | 0.34 | 2 | 0.25 | 0 | 0.00 | 0 | 0.00 | 0 | 0.00 | 36 | 0.44 | 1 | 0.01 | 37 | 0.08 |
| *Promicromonospora* | 0 | 0.00 | 1 | 0.27 | 0 | 0.00 | 1 | 0.35 | 0 | 0.00 | 1 | 0.12 | 0 | 0.00 | 13 | 0.13 | 0 | 0.00 | 1 | 0.01 | 0 | 0.00 | 14 | 0.03 |
| *Pseudomonas* | 10 | 4.37 | 8 | 2.20 | 6 | 2.09 | 8 | 2.83 | 5 | 1.72 | 21 | 2.58 | 43 | 1.17 | 30 | 0.29 | 33 | 0.31 | 67 | 0.82 | 14 | 0.09 | 187 | 0.38 |
| *Pseudonocardia* | 1 | 0.44 | 2 | 0.55 | 1 | 0.35 | 3 | 1.06 | 0 | 0.00 | 6 | 0.74 | 1 | 0.03 | 10 | 0.10 | 1 | 0.01 | 4 | 0.05 | 0 | 0.00 | 16 | 0.03 |
| *Pseudoxanthomonas* | 1 | 0.44 | 2 | 0.55 | 0 | 0.00 | 0 | 0.00 | 0 | 0.00 | 2 | 0.25 | 1 | 0.03 | 20 | 0.20 | 0 | 0.00 | 0 | 0.00 | 0 | 0.00 | 21 | 0.04 |
| *Psychrobacter* | 0 | 0.00 | 0 | 0.00 | 0 | 0.00 | 1 | 0.35 | 1 | 0.34 | 1 | 0.12 | 0 | 0.00 | 0 | 0.00 | 0 | 0.00 | 1 | 0.01 | 1 | 0.01 | 2 | 0.00 |
| *Ralstonia* | 2 | 0.87 | 2 | 0.55 | 0 | 0.00 | 2 | 0.71 | 2 | 0.69 | 2 | 0.25 | 3 | 0.08 | 5 | 0.05 | 0 | 0.00 | 12 | 0.15 | 8 | 0.05 | 28 | 0.06 |
| *Ramlibacter* | 1 | 0.44 | 2 | 0.55 | 0 | 0.00 | 1 | 0.35 | 0 | 0.00 | 3 | 0.37 | 1 | 0.03 | 4 | 0.04 | 0 | 0.00 | 2 | 0.02 | 0 | 0.00 | 7 | 0.01 |
| *Rathayibacter* | 1 | 0.44 | 4 | 1.10 | 1 | 0.35 | 1 | 0.35 | 1 | 0.34 | 4 | 0.49 | 3 | 0.08 | 84 | 0.82 | 6 | 0.06 | 18 | 0.22 | 8 | 0.05 | 119 | 0.24 |
| *Rheinheimera* | 0 | 0.00 | 0 | 0.00 | 1 | 0.35 | 1 | 0.35 | 0 | 0.00 | 2 | 0.25 | 0 | 0.00 | 0 | 0.00 | 2 | 0.02 | 20 | 0.24 | 0 | 0.00 | 22 | 0.04 |
| *Rhizobium* | 3 | 1.31 | 0 | 0.00 | 1 | 0.35 | 0 | 0.00 | 0 | 0.00 | 3 | 0.37 | 78 | 2.13 | 0 | 0.00 | 6 | 0.06 | 0 | 0.00 | 0 | 0.00 | 84 | 0.17 |
| *Rhodanobacter* | 0 | 0.00 | 0 | 0.00 | 0 | 0.00 | 2 | 0.71 | 2 | 0.69 | 3 | 0.37 | 0 | 0.00 | 0 | 0.00 | 0 | 0.00 | 11 | 0.13 | 5 | 0.03 | 16 | 0.03 |
| *Rhodobacter* | 2 | 0.87 | 1 | 0.27 | 1 | 0.35 | 1 | 0.35 | 1 | 0.34 | 2 | 0.25 | 10 | 0.27 | 10 | 0.10 | 19 | 0.18 | 3 | 0.04 | 24 | 0.15 | 66 | 0.13 |
| *Rhodococcus* | 1 | 0.44 | 2 | 0.55 | 1 | 0.35 | 0 | 0.00 | 1 | 0.34 | 3 | 0.37 | 2 | 0.05 | 4 | 0.04 | 2 | 0.02 | 0 | 0.00 | 4 | 0.02 | 12 | 0.02 |
| *Rhodoplanes* | 4 | 1.75 | 3 | 0.82 | 0 | 0.00 | 0 | 0.00 | 3 | 1.03 | 7 | 0.86 | 5 | 0.14 | 7 | 0.07 | 0 | 0.00 | 0 | 0.00 | 13 | 0.08 | 25 | 0.05 |
| *Rhodovulum* | 3 | 1.31 | 0 | 0.00 | 2 | 0.70 | 2 | 0.71 | 1 | 0.34 | 5 | 0.62 | 21 | 0.57 | 0 | 0.00 | 13 | 0.12 | 4 | 0.05 | 6 | 0.04 | 44 | 0.09 |
| *Roseomonas* | 0 | 0.00 | 3 | 0.82 | 3 | 1.05 | 2 | 0.71 | 2 | 0.69 | 4 | 0.49 | 0 | 0.00 | 16 | 0.16 | 15 | 0.14 | 7 | 0.09 | 55 | 0.33 | 93 | 0.19 |
| *Rubellimicrobium* | 0 | 0.00 | 4 | 1.10 | 0 | 0.00 | 1 | 0.35 | 1 | 0.34 | 4 | 0.49 | 0 | 0.00 | 29 | 0.28 | 0 | 0.00 | 4 | 0.05 | 1 | 0.01 | 34 | 0.07 |
| *Rubrivivax* | 0 | 0.00 | 2 | 0.55 | 0 | 0.00 | 0 | 0.00 | 0 | 0.00 | 2 | 0.25 | 0 | 0.00 | 2 | 0.02 | 0 | 0.00 | 0 | 0.00 | 0 | 0.00 | 2 | 0.00 |
| *Rubrobacter* | 0 | 0.00 | 1 | 0.27 | 0 | 0.00 | 0 | 0.00 | 0 | 0.00 | 1 | 0.12 | 0 | 0.00 | 1 | 0.01 | 0 | 0.00 | 0 | 0.00 | 0 | 0.00 | 1 | 0.00 |
| *Ruminococcus* | 4 | 1.75 | 2 | 0.55 | 0 | 0.00 | 2 | 0.71 | 4 | 1.38 | 12 | 1.48 | 6 | 0.16 | 19 | 0.19 | 0 | 0.00 | 4 | 0.05 | 18 | 0.11 | 47 | 0.10 |
| *Saccharothrix* | 0 | 0.00 | 1 | 0.27 | 0 | 0.00 | 0 | 0.00 | 0 | 0.00 | 1 | 0.12 | 0 | 0.00 | 3 | 0.03 | 0 | 0.00 | 0 | 0.00 | 0 | 0.00 | 3 | 0.01 |
| *Salinibacterium* | 1 | 0.44 | 1 | 0.27 | 1 | 0.35 | 1 | 0.35 | 1 | 0.34 | 1 | 0.12 | 91 | 2.48 | 244 | 2.39 | 359 | 3.34 | 95 | 1.16 | 660 | 4.02 | 1449 | 2.94 |
| *Sanguibacter* | 1 | 0.44 | 1 | 0.27 | 1 | 0.35 | 1 | 0.35 | 0 | 0.00 | 1 | 0.12 | 16 | 0.44 | 5 | 0.05 | 1 | 0.01 | 1 | 0.01 | 0 | 0.00 | 23 | 0.05 |
| *SC3-56* | 0 | 0.00 | 1 | 0.27 | 0 | 0.00 | 0 | 0.00 | 0 | 0.00 | 1 | 0.12 | 0 | 0.00 | 1 | 0.01 | 0 | 0.00 | 0 | 0.00 | 0 | 0.00 | 1 | 0.00 |
| *Serratia* | 1 | 0.44 | 3 | 0.82 | 3 | 1.05 | 3 | 1.06 | 4 | 1.38 | 4 | 0.49 | 1 | 0.03 | 68 | 0.66 | 27 | 0.25 | 249 | 3.03 | 48 | 0.29 | 393 | 0.80 |
| *Sharpea* | 0 | 0.00 | 0 | 0.00 | 0 | 0.00 | 0 | 0.00 | 1 | 0.34 | 1 | 0.12 | 0 | 0.00 | 0 | 0.00 | 0 | 0.00 | 0 | 0.00 | 3 | 0.02 | 3 | 0.01 |
| *Shewanella* | 0 | 0.00 | 0 | 0.00 | 0 | 0.00 | 0 | 0.00 | 1 | 0.34 | 1 | 0.12 | 0 | 0.00 | 0 | 0.00 | 0 | 0.00 | 0 | 0.00 | 1 | 0.01 | 1 | 0.00 |
| *Sinomonas* | 2 | 0.87 | 0 | 0.00 | 0 | 0.00 | 2 | 0.71 | 0 | 0.00 | 2 | 0.25 | 5 | 0.14 | 0 | 0.00 | 0 | 0.00 | 17 | 0.21 | 0 | 0.00 | 22 | 0.04 |
| *Skermanella* | 1 | 0.44 | 1 | 0.27 | 0 | 0.00 | 2 | 0.71 | 0 | 0.00 | 2 | 0.25 | 1 | 0.03 | 5 | 0.05 | 0 | 0.00 | 4 | 0.05 | 0 | 0.00 | 10 | 0.02 |
| *Solibacillus* | 0 | 0.00 | 0 | 0.00 | 1 | 0.35 | 1 | 0.35 | 1 | 0.34 | 1 | 0.12 | 0 | 0.00 | 0 | 0.00 | 2 | 0.02 | 1 | 0.01 | 3 | 0.02 | 6 | 0.01 |
| *Sorangium* | 0 | 0.00 | 1 | 0.27 | 0 | 0.00 | 0 | 0.00 | 0 | 0.00 | 1 | 0.12 | 0 | 0.00 | 9 | 0.09 | 0 | 0.00 | 0 | 0.00 | 0 | 0.00 | 9 | 0.02 |
| *Sphaerisporangium* | 1 | 0.44 | 0 | 0.00 | 0 | 0.00 | 0 | 0.00 | 0 | 0.00 | 1 | 0.12 | 1 | 0.03 | 0 | 0.00 | 0 | 0.00 | 0 | 0.00 | 0 | 0.00 | 1 | 0.00 |
| *Sphingobacterium* | 1 | 0.44 | 5 | 1.37 | 6 | 2.09 | 4 | 1.41 | 4 | 1.38 | 9 | 1.11 | 11 | 0.30 | 13 | 0.13 | 12 | 0.11 | 15 | 0.18 | 7 | 0.04 | 58 | 0.12 |
| *Sphingobium* | 4 | 1.75 | 2 | 0.55 | 5 | 1.74 | 3 | 1.06 | 2 | 0.69 | 6 | 0.74 | 30 | 0.82 | 9 | 0.09 | 16 | 0.15 | 42 | 0.51 | 10 | 0.06 | 107 | 0.22 |
| ***Sphingomonas*** | **19** | **8.30** | **45** | **12.36** | **95** | **33.10** | **41** | **14.49** | **81** | **27.93** | **138** | **16.97** | **157** | **4.28** | **1961** | **19.17** | **5013** | **46.62** | **1330** | **16.20** | **9663** | **58.82** | **18124** | **36.77** |
| *Sphingopyxis* | 2 | 0.87 | 1 | 0.27 | 1 | 0.35 | 0 | 0.00 | 0 | 0.00 | 2 | 0.25 | 12 | 0.33 | 1 | 0.01 | 3 | 0.03 | 0 | 0.00 | 0 | 0.00 | 16 | 0.03 |
| *Spirosoma* | 0 | 0.00 | 2 | 0.55 | 3 | 1.05 | 0 | 0.00 | 4 | 1.38 | 5 | 0.62 | 0 | 0.00 | 6 | 0.06 | 8 | 0.07 | 0 | 0.00 | 93 | 0.57 | 107 | 0.22 |
| *Sporosarcina* | 0 | 0.00 | 0 | 0.00 | 0 | 0.00 | 0 | 0.00 | 1 | 0.34 | 1 | 0.12 | 0 | 0.00 | 0 | 0.00 | 0 | 0.00 | 0 | 0.00 | 2 | 0.01 | 2 | 0.00 |
| *Staphylococcus* | 0 | 0.00 | 2 | 0.55 | 2 | 0.70 | 4 | 1.41 | 2 | 0.69 | 5 | 0.62 | 0 | 0.00 | 11 | 0.11 | 2 | 0.02 | 31 | 0.38 | 2 | 0.01 | 46 | 0.09 |
| *Stenotrophomonas* | 3 | 1.31 | 2 | 0.55 | 3 | 1.05 | 2 | 0.71 | 2 | 0.69 | 7 | 0.86 | 40 | 1.09 | 20 | 0.20 | 21 | 0.20 | 167 | 2.03 | 5 | 0.03 | 253 | 0.51 |
| *Steroidobacter* | 0 | 0.00 | 3 | 0.82 | 0 | 0.00 | 3 | 1.06 | 1 | 0.34 | 5 | 0.62 | 0 | 0.00 | 4 | 0.04 | 0 | 0.00 | 11 | 0.13 | 7 | 0.04 | 22 | 0.04 |
| *Streptococcus* | 0 | 0.00 | 2 | 0.55 | 3 | 1.05 | 4 | 1.41 | 4 | 1.38 | 5 | 0.62 | 0 | 0.00 | 11 | 0.11 | 4 | 0.04 | 75 | 0.91 | 12 | 0.07 | 102 | 0.21 |
| *Streptomyces* | 1 | 0.44 | 4 | 1.10 | 1 | 0.35 | 1 | 0.35 | 3 | 1.03 | 6 | 0.74 | 3 | 0.08 | 54 | 0.53 | 2 | 0.02 | 5 | 0.06 | 27 | 0.16 | 91 | 0.18 |
| *Terribacillus* | 1 | 0.44 | 0 | 0.00 | 0 | 0.00 | 0 | 0.00 | 0 | 0.00 | 1 | 0.12 | 1 | 0.03 | 0 | 0.00 | 0 | 0.00 | 0 | 0.00 | 0 | 0.00 | 1 | 0.00 |
| *Thermomonas* | 1 | 0.44 | 0 | 0.00 | 0 | 0.00 | 0 | 0.00 | 0 | 0.00 | 1 | 0.12 | 2 | 0.05 | 0 | 0.00 | 0 | 0.00 | 0 | 0.00 | 0 | 0.00 | 2 | 0.00 |
| *Thermus* | 0 | 0.00 | 0 | 0.00 | 0 | 0.00 | 1 | 0.35 | 0 | 0.00 | 1 | 0.12 | 0 | 0.00 | 0 | 0.00 | 0 | 0.00 | 1 | 0.01 | 0 | 0.00 | 1 | 0.00 |
| *Trabulsiella* | 0 | 0.00 | 1 | 0.27 | 0 | 0.00 | 0 | 0.00 | 1 | 0.34 | 1 | 0.12 | 0 | 0.00 | 1 | 0.01 | 0 | 0.00 | 0 | 0.00 | 1 | 0.01 | 2 | 0.00 |
| *Turicibacter* | 0 | 0.00 | 0 | 0.00 | 0 | 0.00 | 0 | 0.00 | 1 | 0.34 | 1 | 0.12 | 0 | 0.00 | 0 | 0.00 | 0 | 0.00 | 0 | 0.00 | 1 | 0.01 | 1 | 0.00 |
| *Vagococcus* | 0 | 0.00 | 1 | 0.27 | 0 | 0.00 | 1 | 0.35 | 0 | 0.00 | 1 | 0.12 | 0 | 0.00 | 1 | 0.01 | 0 | 0.00 | 1 | 0.01 | 0 | 0.00 | 2 | 0.00 |
| *Virgisporangium* | 0 | 0.00 | 2 | 0.55 | 0 | 0.00 | 0 | 0.00 | 0 | 0.00 | 2 | 0.25 | 0 | 0.00 | 13 | 0.13 | 0 | 0.00 | 0 | 0.00 | 0 | 0.00 | 13 | 0.03 |
| *Wautersiella* | 0 | 0.00 | 0 | 0.00 | 0 | 0.00 | 0 | 0.00 | 1 | 0.34 | 1 | 0.12 | 0 | 0.00 | 0 | 0.00 | 0 | 0.00 | 0 | 0.00 | 3 | 0.02 | 3 | 0.01 |
| *Williamsia* | 1 | 0.44 | 2 | 0.55 | 0 | 0.00 | 0 | 0.00 | 0 | 0.00 | 2 | 0.25 | 18 | 0.49 | 4 | 0.04 | 0 | 0.00 | 0 | 0.00 | 0 | 0.00 | 22 | 0.04 |
| *Wohlfahrtiimonas* | 0 | 0.00 | 0 | 0.00 | 1 | 0.35 | 0 | 0.00 | 0 | 0.00 | 1 | 0.12 | 0 | 0.00 | 0 | 0.00 | 4 | 0.04 | 0 | 0.00 | 0 | 0.00 | 4 | 0.01 |
| *Xanthomonas* | 1 | 0.44 | 0 | 0.00 | 0 | 0.00 | 0 | 0.00 | 0 | 0.00 | 1 | 0.12 | 2 | 0.05 | 0 | 0.00 | 0 | 0.00 | 0 | 0.00 | 0 | 0.00 | 2 | 0.00 |
| *Yersinia* | 1 | 0.44 | 0 | 0.00 | 0 | 0.00 | 0 | 0.00 | 0 | 0.00 | 1 | 0.12 | 2 | 0.05 | 0 | 0.00 | 0 | 0.00 | 0 | 0.00 | 0 | 0.00 | 2 | 0.00 |
| *ZD0117* | 1 | 0.44 | 0 | 0.00 | 0 | 0.00 | 0 | 0.00 | 0 | 0.00 | 1 | 0.12 | 1 | 0.03 | 0 | 0.00 | 0 | 0.00 | 0 | 0.00 | 0 | 0.00 | 1 | 0.00 |
| *Zoogloea* | 0 | 0.00 | 0 | 0.00 | 0 | 0.00 | 1 | 0.35 | 0 | 0.00 | 1 | 0.12 | 0 | 0.00 | 0 | 0.00 | 0 | 0.00 | 1 | 0.01 | 0 | 0.00 | 1 | 0.00 |
| Total | 229 |  | 364 |  | 287 |  | 283 |  | 290 |  | 813 |  | 2057 |  | 6551 |  | 7340 |  | 5497 |  | 13397 |  | 34842 |  |

**Supplementary Table S4.** Distribution of the shared and unique OTUs (at phylum level) in *Stevia* leaf samples at different growth stages

| Phylum | C-0 U | | | | C-2 U | | | | C-4 U | | | | C-0-2 C | | | |
| --- | --- | --- | --- | --- | --- | --- | --- | --- | --- | --- | --- | --- | --- | --- | --- | --- |
|  | OTU | % | Reads | % | OTU | % | Reads | % | OTU | % | Reads | % | OTU | % | Reads | % |
| Proteobacteria | 205 | 60.83 | 345 | 59.18 | 222 | 44.49 | 583 | 31.16 | 376 | 80.51 | 973 | 84.61 | 47 | 51.65 | 408 | 38.13 |
| Actinobacteria | 74 | 21.96 | 128 | 21.96 | 135 | 27.05 | 496 | 26.51 | 50 | 10.71 | 74 | 6.43 | 28 | 30.77 | 376 | 35.14 |
| Bacteroidetes | 19 | 5.64 | 33 | 5.66 | 81 | 16.23 | 398 | 21.27 | 24 | 5.14 | 51 | 4.43 | 4 | 4.40 | 70 | 6.54 |
| Firmicutes | 23 | 6.82 | 56 | 9.61 | 32 | 6.41 | 268 | 14.32 | 11 | 2.36 | 25 | 2.17 | 8 | 8.79 | 178 | 16.64 |
| Gemmatimonadetes | 4 | 1.19 | 4 | 0.69 | 18 | 3.61 | 73 | 3.90 | 4 | 0.86 | 25 | 2.17 | 2 | 2.20 | 28 | 2.62 |
| Acidobacteria | 6 | 1.78 | 9 | 1.54 | 9 | 1.80 | 49 | 2.62 | 2 | 0.43 | 2 | 0.17 | 2 | 2.20 | 10 | 0.93 |
| TM7 | 3 | 0.89 | 3 | 0.51 | 1 | 0.20 | 3 | 0.16 | 0 | 0.00 | 0 | 0.00 | 0 | 0.00 | 0 | 0.00 |
| Planctomycetes | 1 | 0.30 | 1 | 0.17 | 1 | 0.20 | 1 | 0.05 | 0 | 0.00 | 0 | 0.00 | 0 | 0.00 | 0 | 0.00 |
| Chlorobi | 1 | 0.30 | 2 | 0.34 | 0 | 0.00 | 0 | 0.00 | 0 | 0.00 | 0 | 0.00 | 0 | 0.00 | 0 | 0.00 |
| Chloroflexi | 1 | 0.30 | 2 | 0.34 | 0 | 0.00 | 0 | 0.00 | 0 | 0.00 | 0 | 0.00 | 0 | 0.00 | 0 | 0.00 |
| Total | 337 |  | 583 |  | 499 |  | 1871 |  | 467 |  | 1150 |  | 91 |  | 1070 |  |
| Phylum | C-0-4 C | | | | C-2-4 C | | |  | C-0-2-4 C | | | | Total | | | |
|  | OTU | % | Reads | % | OTU | % | Reads | % | OTU | % | Reads | % | OTU | % | Reads | % |
| Proteobacteria | 60 | 84.51 | 462 | 90.06 | 105 | 75.54 | 1073 | 60.52 | 72 | 75.00 | 15052 | 85.72 | 1087 | 63.94 | 19340 | 78.9 |
| Actinobacteria | 7 | 9.86 | 41 | 7.99 | 16 | 11.51 | 481 | 27.13 | 19 | 19.79 | 2418 | 13.77 | 329 | 19.35 | 4284 | 17.5 |
| Bacteroidetes | 1 | 1.41 | 3 | 0.58 | 11 | 7.91 | 181 | 10.21 | 2 | 2.08 | 34 | 0.19 | 142 | 8.35 | 932 | 3.8 |
| Firmicutes | 2 | 2.82 | 5 | 0.97 | 6 | 4.32 | 36 | 2.03 | 2 | 2.08 | 27 | 0.15 | 84 | 4.94 | 659 | 2.7 |
| Gemmatimonadetes | 0 | 0.00 | 0 | 0.00 | 1 | 0.72 | 2 | 0.11 | 0 | 0.00 | 0 | 0.00 | 29 | 1.71 | 168 | 0.7 |
| Acidobacteria | 1 | 1.41 | 2 | 0.39 | 0 | 0.00 | 0 | 0.00 | 1 | 1.04 | 28 | 0.16 | 21 | 1.24 | 118 | 0.5 |
| TM7 | 0 | 0.00 | 0 | 0.00 | 0 | 0.00 | 0 | 0.00 | 0 | 0.00 | 0 | 0.00 | 4 | 0.24 | 8 | 0.0 |
| Planctomycetes | 0 | 0.00 | 0 | 0.00 | 0 | 0.00 | 0 | 0.00 | 0 | 0.00 | 0 | 0.00 | 2 | 0.12 | 4 | 0.0 |
| Chlorobi | 0 | 0.00 | 0 | 0.00 | 0 | 0.00 | 0 | 0.00 | 0 | 0.00 | 0 | 0.00 | 1 | 0.06 | 2 | 0.0 |
| Chloroflexi | 0 | 0.00 | 0 | 0.00 | 0 | 0.00 | 0 | 0.00 | 0 | 0.00 | 0 | 0.00 | 1 | 0.06 | 2 | 0.0 |
| Total | 71 |  | 513 |  | 139 |  | 1773 |  | 96 |  | 17559 |  | 1700 |  | 25517 |  |

**Supplementary Table S5**. Distribution of the core biome of endophytic bacteria (at genus level) in *Stevia* leaf samples at different growth stages

| Genus | C-0 | | C-2 | | C-4 | | F-2 | | F-4 | | Core community | |
| --- | --- | --- | --- | --- | --- | --- | --- | --- | --- | --- | --- | --- |
|  | Reads | % | Reads | % | Reads | % | Reads | % | Reads | % | Reads | % |
| *Sphingomonas* | 157 | 4.28 | 1961 | **19.17** | 5013 | **46.62** | 1330 | **16.20** | 9663 | **58.82** | 18124 | **36.77** |
| *Methylobacterium* | 174 | 4.74 | 1686 | **16.48** | 1063 | 9.89 | 1669 | **20.33** | 1137 | 6.92 | 5729 | **11.62** |
| *Salinibacterium* | 91 | 2.48 | 244 | 2.39 | 359 | 3.34 | 95 | 1.16 | 660 | 4.02 | 1449 | 2.94 |
| *Escherichia* | 2 | 0.05 | 108 | 1.06 | 20 | 0.19 | 598 | 7.28 | 127 | 0.77 | 855 | 1.73 |
| *Agrobacterium* | 454 | **12.37** | 34 | 0.33 | 142 | 1.32 | 10 | 0.12 | 49 | 0.30 | 689 | 1.40 |
| *Acinetobacter* | 111 | 3.02 | 99 | 0.97 | 28 | 0.26 | 61 | 0.74 | 315 | 1.92 | 614 | 1.25 |
| *Erwinia* | 267 | **7.28** | 64 | 0.63 | 215 | 2.00 | 15 | 0.18 | 31 | 0.19 | 592 | 1.20 |
| *Hymenobacter* | 0 | 0.00 | 190 | 1.86 | 30 | 0.28 | 68 | 0.83 | 248 | 1.51 | 536 | 1.09 |
| *Nocardioides* | 20 | 0.54 | 271 | 2.65 | 8 | 0.07 | 101 | 1.23 | 48 | 0.29 | 448 | 0.91 |
| *Kineococcus* | 3 | 0.08 | 187 | 1.83 | 23 | 0.21 | 112 | 1.36 | 71 | 0.43 | 396 | 0.80 |
| *Geodermatophilus* | 6 | 0.16 | 121 | 1.18 | 38 | 0.35 | 45 | 0.55 | 184 | 1.12 | 394 | 0.80 |
| *Serratia* | 1 | 0.03 | 68 | 0.66 | 27 | 0.25 | 249 | 3.03 | 48 | 0.29 | 393 | 0.80 |
| *Novosphingobium* | 131 | 3.57 | 8 | 0.08 | 44 | 0.41 | 2 | 0.02 | 157 | 0.96 | 342 | 0.69 |
| *Stenotrophomonas* | 40 | 1.09 | 20 | 0.20 | 21 | 0.20 | 167 | 2.03 | 5 | 0.03 | 253 | 0.51 |
| *Pseudomonas* | 43 | 1.17 | 30 | 0.29 | 33 | 0.31 | 67 | 0.82 | 14 | 0.09 | 187 | 0.38 |
| *Exiguobacterium* | 0 | 0.00 | 163 | 1.59 | 0 | 0.00 | 13 | 0.16 | 0 | 0.00 | 176 | 0.36 |
| *Bacillus* | 53 | 1.44 | 34 | 0.33 | 1 | 0.01 | 32 | 0.39 | 3 | 0.02 | 123 | 0.25 |
| *Spirosoma* | 0 | 0.00 | 6 | 0.06 | 8 | 0.07 | 0 | 0.00 | 93 | 0.57 | 107 | 0.22 |
| *Streptococcus* | 0 | 0.00 | 11 | 0.11 | 4 | 0.04 | 75 | 0.91 | 12 | 0.07 | 102 | 0.21 |
| *Rhizobium* | 78 | 2.13 | 0 | 0.00 | 6 | 0.06 | 0 | 0.00 | 0 | 0.00 | 84 | 0.17 |
